# Multi-Tissue Microarray Analysis Identifies a Molecular Signature of Regeneration

**DOI:** 10.1371/journal.pone.0052375

**Published:** 2012-12-26

**Authors:** Sarah E. Mercer, Chia-Ho Cheng, Donald L. Atkinson, Jennifer Krcmery, Claudia E. Guzman, David T. Kent, Katherine Zukor, Kenneth A. Marx, Shannon J. Odelberg, Hans-Georg Simon

**Affiliations:** 1 Department of Pediatrics, Northwestern University, Feinberg School of Medicine and Children’s Memorial Research Center, Chicago, Illinois, United States of America; 2 Department of Chemistry, University of Massachusetts-Lowell, Lowell, Massachusetts, United States of America; 3 Department of Internal Medicine, Division of Cardiology, University of Utah, Salt Lake City, Utah, United States of America; Shriners Hospitals for Children, United States of America

## Abstract

The inability to functionally repair tissues that are lost as a consequence of disease or injury remains a significant challenge for regenerative medicine. The molecular and cellular processes involved in complete restoration of tissue architecture and function are expected to be complex and remain largely unknown. Unlike humans, certain salamanders can completely regenerate injured tissues and lost appendages without scar formation. A parsimonious hypothesis would predict that all of these regenerative activities are regulated, at least in part, by a common set of genes. To test this hypothesis and identify genes that might control conserved regenerative processes, we performed a comprehensive microarray analysis of the early regenerative response in five regeneration-competent tissues from the newt *Notophthalmus viridescens*. Consistent with this hypothesis, we established a molecular signature for regeneration that consists of common genes or gene family members that exhibit dynamic differential regulation during regeneration in multiple tissue types. These genes include members of the matrix metalloproteinase family and its regulators, extracellular matrix components, genes involved in controlling cytoskeleton dynamics, and a variety of immune response factors. Gene Ontology term enrichment analysis validated and supported their functional activities in conserved regenerative processes. Surprisingly, dendrogram clustering and RadViz classification also revealed that each regenerative tissue had its own unique temporal expression profile, pointing to an inherent tissue-specific regenerative gene program. These new findings demand a reconsideration of how we conceptualize regenerative processes and how we devise new strategies for regenerative medicine.

## Introduction

The regenerative ability of mammalian tissues is typically very limited, with the default response to damage characterized by scar formation [1]. In contrast, the red-spotted newt *Notophthalmus viridescens* possesses an astonishing capacity to regenerate damaged limbs, tails, lenses, and significant portions of the heart and central nervous system with nearly complete functional recovery [2]. These remarkable abilities have made the newt a lasting model organism in regenerative biology, and research in this and related salamander species has produced substantial information on the molecular and cellular activities involved in the regenerative response [3]–[8]. Collective experimental evidence suggests that tissue regeneration centers on the reversal of the cells’ differentiation status at the injury site, enabling the proliferative and migratory cell responses necessary for functional tissue replacement [9]–[12]. However, the molecular mechanisms underpinning these cellular activities and tissue responses have remained largely elusive, as genetic manipulation in salamanders is currently not at a level comparable to other model organisms. As an alternative strategy, we have employed a novel molecular approach by combining differential display and custom microarrays for a comprehensive screen aimed at identifying and characterizing concerted gene activities in the regenerative response of five different regeneration-competent tissues: forelimb, hindlimb, tail, heart, and spinal cord (both local spinal cord tissue and brain following spinal cord transection). To identify the specific gene programs that drive the onset and progression of regeneration in these organs, our analysis focused on the regenerative response within the initial three weeks after injury.

Previous microarray studies with regeneration-competent vertebrates, including *Xenopus*, zebrafish, medaka fish, and several salamander species provided important insights into regenerative response genes [13]–[24]. However, these studies were limited by their focus on the regeneration of a single tissue type over only a few time points or their comparison of mutant or regeneration-incompetent conditions to productive tissue regeneration. Additionally, these investigations typically considered a large initial gene set, with subsequent analytical effort primarily devoted to the discovery of those genes showing high levels of differential expression. In contrast to these earlier approaches, we developed Agilent microarrays containing oligonucleotides largely derived from the sequences of genes identified in previous mRNA differential display screens of regenerating newt limbs, tails and brain tissue collected during spinal cord regeneration performed by the Simon and Odelberg laboratories (described in [25]–[27]). The pre-selection for cDNAs enriched in regenerative programs and the high number of replicates placed on our custom arrays allowed for substantial qualitative and accurate quantitative analyses that have not been achieved previously. Moreover, by including multiple tissue types over an extensive time frame, we were able to develop a deep and unprecedented understanding of the overarching signature of genes driving regenerative processes. Figure S1 outlines the experimental design and the steps employed in our analyses.

In addition to discovering conserved molecular programs of regeneration embedded within the temporal developmental sequence, our approach identified gene activities specific to each of the regenerating tissue types. This finding provides the new perspective that temporal and spatial gene activities are intertwined and orchestrated in context to rebuild a particular tissue. The results presented here should greatly aid researchers in developing testable hypotheses concerning the common molecular mechanisms that control all regenerative processes, as well as those that control tissue-specific regeneration.

## Results and Discussion

### What are the Molecular Programs that Define Vertebrate Tissue Regeneration?

In order to determine a molecular signature of regeneration, we selected five different newt tissues that are competent to undergo regenerative repair, including the fore- and hindlimbs, tail, heart, and spinal cord (both local spinal cord tissue and brain following spinal cord transection). In addition, to understand the sequence of gene activities during the induction and progression of regenerative processes, we examined changes in gene expression in each tissue type over a time course starting as early as 1 day postamputation (dpa) and spanning the following three weeks after injury, such that the majority of investigated tissues had five associated temporal data sets. Importantly, by utilizing both multiple tissue types and time points, we also provided a significant regulatory component to our investigation.

**Figure 1:**
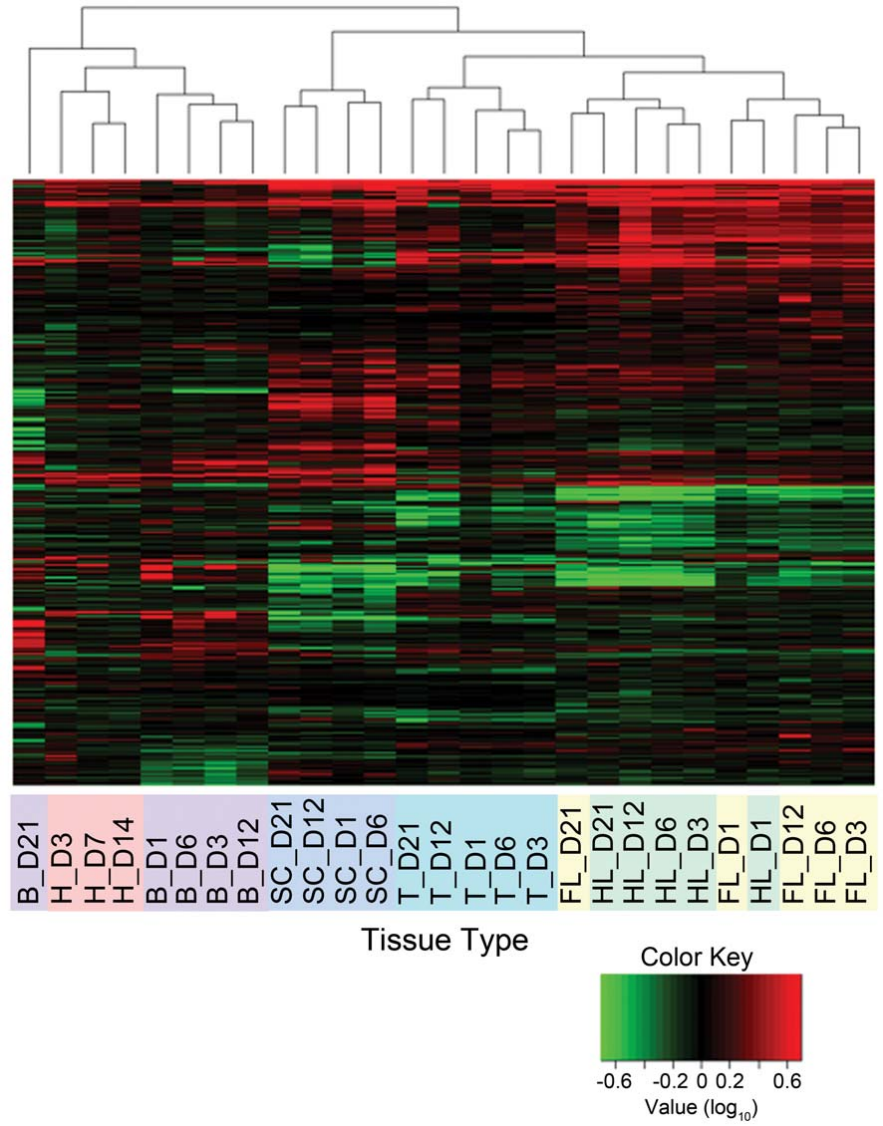
Gene expression profiling of multiple tissue types reveals regeneration-specific molecular activities. A total of 1,346 genes exhibited ≥2-fold differential expression in at least one tissue type for at least one time point during the first 3 weeks of regeneration. These genes were hierarchically clustered into groups based on similarity of their expression patterns across the evaluated time points. Differentially expressed genes were assigned a color intensity with a 5-fold bound color key (log_10_ scale). Red and green represent increased and decreased expression, respectively. Regenerating tissues are color-coded and abbreviated as: B = brain following spinal cord transection; H = heart; SC = spinal cord; T = tail; FL = forelimb; HL = hindlimb. Days postamputation are indicated following tissue type designation.

The microarrays probed 1876 genes, with over 90% of these sequences derived from mRNA differential display screens of regenerating newt limbs, tails and brain tissue collected during spinal cord regeneration [25]–[27], while the remaining probes represented all other newt sequences available from GenBank at the time of array design. Unlike other approaches, this array customization allowed for a significant enrichment of regeneration-specific gene activities. Heart tissues were not included in the initial differential display enrichment step and it is possible that a few genes exclusive to the heart are missing. However, we found that most genes are expressed in multiple tissue types, and heart tissue frequently exhibited expression profiles comparable to regenerating limbs, tails and spinal cords for the genes present on the arrays (Figure S2 and Dataset S1). Considering all tissue types and time points, we found 1346 genes with ≥2-fold changes in expression levels relative to the corresponding unamputated controls (Figure 1). We credit the identification of such a robust number of differentially expressed genes to the pre-selection of cDNAs printed on our array slides, which allowed for the identification of a large, regeneration-specific gene set with little to no data loss due to background interference (full array data available in Dataset S1).

The majority of differentially expressed genes (71.6%) exhibited between 2- and 5-fold up- or down-regulation for at least one time point, though a large number of genes (179 genes, 9.6% of total) showed ≥10-fold differential expression for at least one time point during regeneration (Table 1). Such marked differential expression may indicate the importance of these specific genes to regeneration (Table 2). Differential activity was in large part equally distributed between up- and down-regulation of expression, though gene upregulation was marginally more common (Table 1 and Figure S2). Importantly, the observed downregulation could be secondary to upregulation of transcriptional repressors. It is also significant to note that the large number of downregulated genes suggests that the loss of activity plays significant roles in the induction and maintenance of regeneration – a concept that is currently underexplored in regenerative biology. In particular, our data reveal a surprisingly high correlation in the specific numbers of up- and down-regulated genes over the regenerative time course across tissue types (Figure S2).

**Table 1:** Summary statistics for regeneration microarray studies.

GENES	UPREGULATION			DOWNREGULATION		
	>2-fold	>5-fold	>10-fold	>2-fold	>5-fold	>10-fold
**All**	1022	251	97	876	215	82
**Percent** **Total**	54.9%	13.5%	5.2%	47.1%	11.6%	4.4%
**Selected**	87	50	26	82	37	23
**Percent** **Total**	4.7%	2.7%	1.4%	4.4%	2.0%	1.2%

Analysis is based on log_10_ transformed data for all tissue types and time points. Each regulation category represents the entire, inclusive data set with percent values derived from dividing the number of genes in each category by the total 1,860 genes represented on the array. Selected differentially expressed genes correlate to the subset of genes and/or gene families that are specifically discussed in the text.

**Table 2:** Conserved molecular programs support regeneration across multiple tissue types.

GeneS	TissueS					
	Forelimb	Hindlimb	Tail	S. Cord	Brain	Heart
***Immune Response***						
**uPAR**	**▴▾**	**▴▾**	**▴**	**▴**	**▴**	**▴▾**
**Apple 4**	**▴**	**▴**	**▴**	**▴**	**▴**	**▴**
**Elafin-like 1**	**▴**	**▴**	**▴**	**▴**		**▴**
**Thrombin**	**▾**		**▴**	**▴**	**▾**	**▴**
**CD3**	**▾**	**▴**		**▾**	**▾**	**▾**
**CXC chemokine**	**▴▾**	**▴**	**▴**	**▾**	**▴▾**	**▾**
**Annexins**	**▴**	**▴**	**▴**	**▾**	**▴▾**	**▾**
**Chromogranin B**		**▾**	**▾**	**▾**	**▾**	**▾**
**Galectins**	**▴▾**	**▴▾**	**▴▾**	**▾**	**▴▾**	
**Phospholipase A2**	**▴▾**		**▾**	**▾**	**▾**	
***Pattern Regulation***						
**Prod-1**	**▴**	**▴**	**▾**	**▴**	**▴**	**▴**
**Msx1**		**▴**	**▴**	**▾**	**▾**	**▴**
**HoxC13**	**▴**	**▴**		**▴**	**▴**	
**HoxC12**	**▴**		**▴**	**▴**		
**HoxC5**	**▴**		**▾**	**▾**	**▾**	
**HoxD10**	**▴▾**	**▴▾**		**▾**		
**HoxC6**		**▾**	**▾**	**▴**	**▴▾**	
**HoxA9**	**▾**	**▾**		**▾**		
**Goosecoid**	**▾**	**▾**	**▾**	**▴**		**▴**
***Tissue Remodeling***						
**Collagens**	**▾**	**▴▾**	**▴▾**	**▴**	**▴▾**	**▴**
**Fibrosin-1**	**▴▾**	**▴**	**▴**	**▴**	**▴**	**▾**
**MMPs**	**▴▾**	**▴**	**▴▾**	**▴**	**▴**	**▴**
**TiMP**	**▴**	**▴**	**▴**	**▴**	**▴**	**▴**
**Fibulin 2**		**▴**	**▴**	**▴**	**▴**	**▴**
**Cathepsin L**	**▴**	**▴**	**▴**	**▴**		**▴**
**Tenascin**	**▴**	**▴**	**▴**	**▴**		**▴**
**Fibronectin**	**▾**	**▴**	**▴**	**▴**		**▴**
***Cytoskeletal Rearrangements***						
**Keratins**	**▴▾**	**▴▾**	**▴▾**	**▴▾**	**▴▾**	**▴▾**
**Genghis Khan**	**▴**	**▴**	**▴▾**	**▴**	**▴**	**▴**
**EP-Cadherin**			**▴**	**▴▾**	**▴**	
**Thymosin β4**	**▴**	**▴**		**▴**	**▴**	
**Vimentin 4**	**▴**	**▴**	**▴**	**▴**		
**Integrins**		**▴**	**▴**	**▴**		
**Tubulins**		**▴**	**▴**	**▴▾**	**▾**	**▴**
***Signaling Cascades***						
**ARL11**	**▴**	**▴**	**▴**	**▴**		**▴**
**Rab 11a**		**▴**	**▴**	**▴**		
**G protein Rad**	**▴**	**▴**	**▴**	**▴**	**▾**	
**FGFs**	**▾**	**▾**			**▾**	**▾**
**FGF receptors**	**▾**	**▾**			**▾**	

Select annotated genes with differential expression during early regeneration were grouped according to their primary *in vivo* function. Upregulation for at least one time point in the designated tissue is indicated by ▴ (≥2-fold), ▴ (≥5-fold), and ▴ (≥10-fold). Downregulation for at least one time point in the designated tissue is indicated by ▾ (≥2-fold), ▾ (≥5-fold), and ▾(≥10-fold). Up- and down-ward arrowheads in a given tissue column for the same gene indicate changes in expression direction over the investigated time course.

### Microarray Validation

Of the 1876 genes represented on the arrays, we recovered high quality data for 1860 genes (over 99%). As each Agilent chip contained between 7 and 23 replicate printings of the oligonucleotide set, we determined that the average standard deviation activity level for each gene was only 2–3% of the gene’s activity level [28]. These data demonstrate the success of our hybridization and signal detection. To further confirm the fidelity of the performance of the microarrays, we conducted quantitative (q) RT-PCR for 13 selected genes using independent biological samples for regenerating forelimb, hindlimb, tail and heart tissues (Table S1). This qRT-PCR analysis supported the array expression data and confirmed the high level of precision and reproducibility of our analysis. In line with current biostatistical observations regarding qRT-PCR validation of microarrays [29], we find that our qRT-PCR data demonstrate a more dynamic maximal range of differential expression. Additional independent qRT-PCR validations on 23 selected genes from our microarrays for forelimb regenerates were previously reported [27]. To further validate our microarray results, we also performed *in situ* hybridizations for a newt *cxcl* chemokine in regenerating fore- and hind-limbs, as well as *mmp9* in regenerating brain and spinal cord tissues. As expected, these *in situ* analyses revealed a substantial upregulation of *cxcl* in regenerating limbs (Figure S3) and *mmp9* in regenerating spinal cord lesions (Figure S4), respectively.

### Immune Response

Injury with tissue loss is a traumatic event that invariably induces an inflammatory response. Accordingly, our data indicate immune system activation early in the regenerative response. While previous reports have noted the importance of immunological activities in regeneration [30], [31], we have identified multiple novel immune response genes with differential expression during regeneration. Furthermore, as a result of our integrated approach, an overarching theme emerged suggesting that pro- and anti-inflammatory signals are finely balanced during tissue regeneration, which is likely an important determinant in scarless wound healing.

A vital component of the plasminogen activation pathway, the urokinase receptor uPAR is generally upregulated across all tissue types during regeneration (Table 2). The plasminogen system has long been implicated in clot lysis and classic wound healing, with uPAR originally thought to support the migration of cells at wound sites by converting urokinase plasminogen to the serine proteinase plasmin at the leading edge of cells, providing for cellular mobility and the degradation of cell debris and extracellular matrices [32]. However, several studies demonstrated that uPAR interacts with a number of other proteins to elicit a variety of cellular responses, including cell differentiation and proliferation – processes vital to regenerative success [33]–[36]. Among these functional interactions, ligand-activated uPAR has been shown to influence integrin-dependent cell-matrix adhesion, migration and proliferation [37]–[39]. Interestingly, our data indicate that the vitronectin-binding α_v_ integrin subunit (GenBank accession number X81108), a recognized binding partner of uPAR, is upregulated in appendage and nervous tissues during regeneration. As integrins can influence a number of intracellular processes through cytoskeletal rearrangements and signal transduction pathways, we speculate that uPAR and its potential binding partner may play multiple roles in the induction and/or maintenance of the regenerative response by supporting cell proliferation, migration and alterations in differentiation status.

Similar to the upregulation of uPAR, the gene we refer to as *apple 4* is also upregulated in all regenerating tissues. This newt cDNA maintains significant sequence homology to both *factor XI* of the coagulation cascade and *prekallikrein* of the kallikrein-kinin system. When prekallikrein is cleaved by factor XIIa, the two resulting peptides, a heavy chain and a light chain, are united by a disulfide bond to form kallikrein. The heavy chain consists of four apple domains that bind to kallikrein’s substrate, kininogen, while the light chain contains the serine protease catalytic domain that cleaves kininogen to form bradykinin [40], a major factor in eliciting the inflammatory response and pain following injury. However, the newt gene is novel in that it contains only the four apple domains and lacks the catalytic serine protease domain. This suggests that the protein encoded by *apple 4* might act as a dominant negative by binding to kininogen and preventing kallikrein-mediated production of bradykinin. We postulate that reduced levels of bradykinin could lead to attenuation in the inflammatory response and possibly a reduction in pain following a serious injury in the newt. In contrast to mammals, the inflammatory response in the regenerating newt is limited and does not lead to scar formation, a process thought to act as a barrier to regeneration [27], [41]. Therefore, it is interesting to speculate that this novel newt gene may be playing a role in both preventing fibrosis and reducing pain during the regenerative process.

### Tissue Remodeling

Functional restoration of a lost structure or organ requires extensive remodeling of the tissue that remains following injury. A significant portion of this process is the clearing of cell and extracellular debris, and our data indicate that a variety of proteases are involved in this process.

Matrix metalloproteinases (MMPs) are dramatically upregulated over the regenerative time course, playing substantial roles in remodeling the extracellular environment of regenerating tissues [27], [42]. Our analysis reveals that mmp-3/mmp-10 (stromelysins; GenBank accession number AY857754) and mmp-9 (gelatinase; GenBank accession number AY857752), widely distributed proteases that play diverse roles in development and disease, are induced early in the regenerative response. Additionally, the collagenase mmp-13, largely considered active only in cartilage and bone [43], [44], is robustly upregulated in all tissue types during regeneration, suggesting broader functions for this enzyme in the regeneration-competent newt. However, this MMP-mediated extracellular remodeling is balanced by the upregulation of tissue inhibitors of MMPs (TIMPs; Table 2). In particular, our data indicate that *timp-1* (GenBank accession number DQ286430) is highly expressed in all regenerating tissues throughout the investigated time window. Timp-1 is an enzyme produced by a variety of cell types, functioning as a specific inhibitor of MMPs including interstitial collagenases, gelatinases and stromelysins [45], [46]. In addition to this classic MMP regulatory role, timp-1 has also been found to inhibit apoptosis and stimulate cell growth [45]–[47]. Therefore, timp-1 may not only temporally and locally modulate the activity of MMPs in regenerating tissues to prevent their excessive proteolytic activity, but may also elicit cell growth/proliferation for regenerative repair [46].

Coupled with the extensive activity of MMPs in the regenerative process, we demonstrate an upregulation of a variety of extracellular matrix (ECM) components, including fibronectin (FN; GenBank accession number S76886) and tenascin-C (TNC; GenBank accession number M76615), matrices critical to development and wound healing across multiple tissue types (Table 2). In fact, our work with regenerating newt forelimbs revealed at the RNA and protein level that FN and TNC, in addition to hyaluronic acid, form a transitional, regeneration-specific matrix capable of inducing regenerative cell behaviors including cell dedifferentiation, proliferation and migration [42], [48]. Beyond the limbs, our array results suggest that similar matrix components function in a number of regenerating tissues, providing deeper information about the conservation of extracellular remodeling as a driving force in regenerating tissues.

### Cytoskeletal Rearrangements

Regenerative repair necessarily involves dramatic changes in tissue architecture concomitant with changes in cell behaviors, as cells must migrate, proliferate and alter their differentiation status. These alterations in cellular activities likely involve the remodeling of subcellular architecture, and our array data suggest distinct cytoskeletal rearrangements in nearly all regenerating tissue types.

The expression profiles demonstrate a substantial and largely conserved upregulation of keratins, vimentins and tubulins (Table 2). Though widely researched as intracellular mechanical scaffolds, much remains to be discovered about the dynamic roles intermediate filaments (IFs) and microtubules have in shaping tissues [49], [50]. In a regenerative context, it is intriguing to consider a regulatory function for these cytoskeletal components in cell proliferation and/or migration. Keratin filaments attach to cell-cell adherens junctions, as well as cell-ECM adhesion sites. In this way, they provide a mechanical scaffold that allows the cell to be highly responsive to its environment. For example, keratins play important roles in cell migration, particularly by influencing changes in cell shape [51]. Similarly, vimentin, the predominant IF in mesenchymal cells, may function primarily in support of the cytoarchitecture. Interestingly, the coexpression of keratin and vimentin can dramatically increase the migratory capacity of a number of cell types [52], [53]. Therefore, the dynamic induction of both these IFs during regeneration suggests an increase in migratory capacity. Our data also reveal an upregulation of tubulin, the basic component of all microtubules (Table 2). Key components of the cytoskeleton, microtubules are long filamentous polymers critical in the development and maintenance of cell shape and cellular transport, with additional functions in cell signaling, cell division and mitosis [54], [55]. During tissue regeneration, an increase in microtubule activity across multiple tissue types suggests an active role in the cell proliferative response.

In concert with this dramatic reorganization of the IF and microtubule cytoarchitecture, actin-based components of the cytoskeleton are dynamically regulated in most regenerating tissues. For example, genghis khan is a protein kinase thought to be an effector of CDC42 for its regulation of actin polymerization. Genghis khan is required for the integrity of the actin cytoskeleton by inhibiting *de novo* actin polymerization [56]. The increased expression of *genghis khan* during tissue regeneration suggests a dramatic change in actin cytoskeleton dynamics. In support of this notion, *thymosin β4* is also upregulated during regeneration and could further refine actin polymerization processes (Table 2). Thymosin β4 sequesters G-actin monomers, a process implicated in the reorganization of actin-based cytoarchitecture that is necessary to support cell migration [57]. β-thymosins are also known to have hormone-like properties. In this context, we note that thymosin β4 has been reported to prime epicardial progenitor cells for transdifferentiation into new cardiomyocytes after myocardial infarction [58], and, along with GATA4, MEF2C and TBX5, can reduce scarring and aid in cardiac function [59].

Collectively, the differential regulation of genes involved in IF, microtubule and actin dynamics suggest dramatic alterations to the cytoskeletal organization of cells undergoing regenerative activities. These cytoskeletal rearrangements may allow for cell shape changes that are required for and possibly drive proliferation, migration and dedifferentiation – processes essential for regenerative repair. Moreover, these cytoskeletal rearrangements correlate with increased integrin expression and significant changes in ECM composition, indicating dynamic signaling between extra- and intra-cellular environments throughout early regeneration (Table 2).

### Pattern Regulation

Regenerative tissue repair involves recognition of tissue loss or injury, followed by complete reconstruction or restoration of the relevant structure. Regeneration in adult animals is often viewed as an example of postembryonic morphogenesis – a recapitulation of development. However, unlike developmental processes, regeneration must build new parts upon existing structures, replacing what was lost in a seamless integration. Therefore, we looked at tissue patterning, considering genes re-utilized from developmental processes as well as those that may maintain regeneration-specific activities.

*Hox* genes are widely recognized for their roles in providing positional information during tissue patterning in the embryo [60]. As expected, our array screen provides evidence for up- or downregulation of multiple *hox* genes in all regenerating tissues tested except the heart (Table 2). This finding is consistent with previous reports that these developmental genes play critical roles in regenerative patterning of the limbs [61]–[63].

During limb regeneration, the blastema gives rise to structures precisely distal to its site of origin, such that the progenitor cells possess a memory of their level of origin along the proximodistal (PD) axis [3]. This PD identity is at least partially encoded by prod-1 (GenBank accession number EU128720), a member of the three-finger protein (TFP) superfamily [64]. In the intact and regenerating limb, prod-1 exists in a PD gradient, and forced misexpression during regeneration can alter the positional identity of forelimb blastema cells from distal to more proximal values [64], [65]. As expected, we find *prod-1* significantly upregulated during fore- and hindlimb regeneration, supporting the earlier conclusion that prod-1 is a cue for local cell identity in the limb. Of note, we also saw *prod-1* dramatically upregulated during heart and spinal cord regeneration (Table 2). This is an interesting finding because in the limbs prod-1 is known to interact with the anterior gradient protein family member n(ewt)AG, a ligand produced by nerve Schwann cells and glands in the wound epidermis [66]. The binding of nAG by prod-1 promotes cell division and our new data suggest a more general and conserved role for the maintenance of positional identity and/or regenerative growth in a variety of regenerating tissue types.

### Signaling Cascades

As discussed above, the regenerative response involves dramatic changes to the composition and distribution of the ECM, as well as substantial modifications to intracellular architecture in regenerating tissues. To better understand the signaling interactions that may influence and ultimately connect these synchronous intracellular and extracellular regenerative processes, we investigated a number of genes involved in signaling cascades.

Fibroblast growth factors (FGFs) are members of a large family of short polypeptides that mediate a variety of biological responses, including cell proliferation, migration and differentiation [67]. FGFs are typically released into the extracellular space where they bind heparan sulphate proteoglycans and subsequently undergo high affinity interactions with one or more of four transmembrane tyrosine kinase receptors (FGFRs). Research efforts in mammalian models have shown that FGF family members play active roles in wound healing, tissue repair and angiogenesis [68]–[70]. We were surprised to find FGFs (*FGF1*, *FGF2* and *FGF4*; GenBank accession numbers AB175665, AB064664 and U76998, respectively) and their receptors (*FGFR1*, *FGFR2* and *FGFR4*; GenBank accession numbers L19868, L19869 and X65059, respectively) downregulated in the majority of regenerating tissues investigated. However, as FGFs are secreted into the extracellular environment, where they typically bind to heparan sulphate proteoglycans, the ECM essentially becomes a local reservoir for FGF signaling [71]. As the extracellular space undergoes extensive remodeling during early tissue regeneration, we speculate that FGFs may be mobilized from this reservoir during the initial phase of regeneration. It may also be possible that FGFs and their receptors are expressed in cell subpopulations and/or limited tissue locations during the early regenerative response, such that we see an overall downregulation in the RNA sample utilized in our array expression analysis. This explanation seems plausible, as previous studies on FGF expression in urodele limb and lens regeneration concluded that enhanced signaling is occurring largely through *in situ* hybridizations or pharmaceutical intervention [72]–[75], such that these disparate methodologies could have produced different results. In addition, previous reports have utilized alternative time points, different species and investigated different members of the FGF family [73], [74], [76]. Therefore, alternative approaches will be necessary in the future to clarify these discrepancies in FGF expression and activity during the induction of vertebrate regeneration.

The Ras superfamily represents evolutionarily conserved classes of genes that typically encode small GTPases that function as molecular switches and signal mediators. ARL11 (also known as ARLTS1, ADP-ribosylation factor-like tumor suppressor gene 1) is a member of the ARF subfamily and recent research strongly indicates that it functions as a tumor suppressor, participating in pro-apoptotic and programmed cell death pathways [77], [78]. Given the somewhat antithetical relationship between cancer and regeneration [79], [80], we were intrigued to see *ARL11* upregulated in nearly every regenerating tissue investigated. This discovery suggests that the tumor suppressor activity of ARL11 may play a critical role during the onset of tissue regeneration, when blastema formation relies heavily on the tight control of progenitor cell generation and propagation. Indeed, recent work in both *Xenopus* and hydra models of regeneration has shown that apoptotic activity is critical in the establishment of the early regenerative response [81], [82]. Therefore, in the context of natural regeneration, ARL11 may support the induction of necessary apoptotic pathways and prevent uncontrolled cell proliferation.

### Do Distinct Molecular Programs Exist in Different Regenerating Tissues?

Multiple tissues in the newt are capable of complete and functional regeneration. We identified common gene activities over a range of regenerating tissues, indicative of conserved, regeneration-specific gene programs. However, our multi-tissue approach also offered the unique advantage of identifying equally important tissue-specific expression profiles. For these analyses, we focused on comparing the regeneration of tissues with similar structure and/or function, including cardiac muscle vs. skeletal muscle and brain vs. spinal cord vs. tail, as well as the inherently different tissue groups–internal organs vs. appendages–in order to extract the most information about tissue specificity in regeneration.

**Figure 2:**
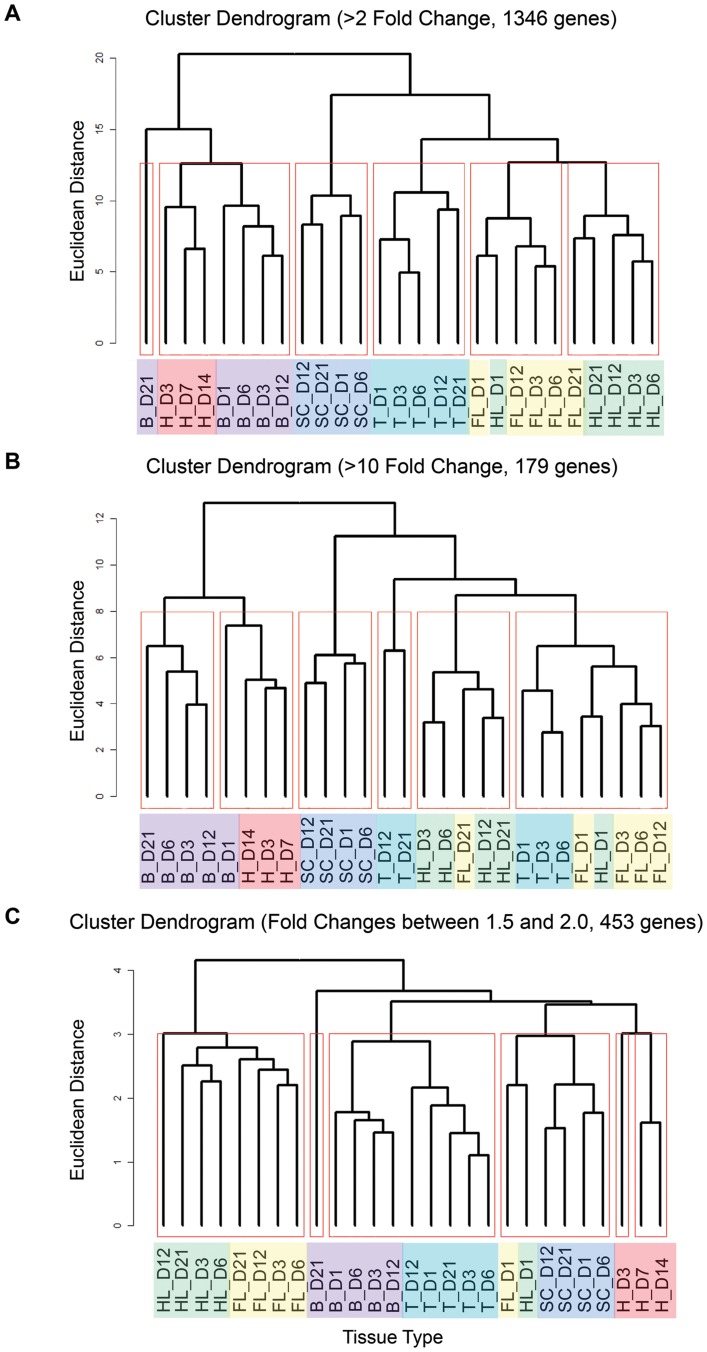
Hierarchical clustering indicates that different tissue types employ distinct gene activities during the regenerative process. A blind clustering algorithm was applied to differentially expressed genes across all tissue types and time points during the initial three weeks of the regenerative response. In the cluster dendrogram representation, regenerating tissues that share similar gene expression profiles will have closer Euclidean distances to common branch points and be grouped into a cluster. When considering those genes with ≥2-fold differential expression (**A**), each regenerating tissue type self-sorts into clusters independent of time point. This clustering of tissue types persists at extremely high (≥10) and low (between 1.5 and 2) fold change measurements (**B** and **C**, respectively), suggesting that each regenerating structure employs tissue-specific gene programs during the induction and early maintenance of regeneration. Regenerating tissues are color-coded and abbreviated as: B = brain following spinal cord transection; H = heart; SC = spinal cord; T = tail; FL = forelimb; HL = hindlimb. Days postamputation are indicated following tissue type designation.

The gene activities present in each regenerating tissue are unique enough to segregate the entire data set by tissue type. By comparing the similarity of all gene expression values among those genes exhibiting differential expression, we find that nearly all time points for each tissue type preferentially group together in a cluster dendrogram (Figure 2). We have high confidence in these dendrograms because our custom arrays provided exceptionally high fidelity data sets due to the high number of replicate printings for each oligonucleotide. The only exception to this self-sorting behavior is found in the expression profiles of the forelimb and hindlimb, which is likely due to the fact that these two appendages are very similar to each other, resulting in co-segregation (Figure 2). Of note, within these mixed clusters, the forelimb and hindlimb data sets frequently sort based on the temporal regenerative sequence, such that the earliest (day 1) and latest (day 21) evaluated time points cluster together independent of limb type, suggesting that the molecular activities in regenerating forelimbs and hindlimbs are exceedingly similar and follow a comparable time course.

It is interesting to consider those few genes with divergent behaviors in fore- versus hindlimbs. One such gene is *cd3,* an immune response gene (Table 2). *cd3* differential expression levels vary between the hindlimb and forelimb. However, the general pattern of differential expression is conserved (Dataset S1). While displaying differences in the levels of differential expression, the same conserved trends in expression over time hold true for other genes, including *rab11a* and *fibronectin*. We speculate that these differences result from the size inequality between the limbs and our tissue harvest methodology, though we cannot rule out that these disparities serve a particular biological function.

It is also noteworthy that the self-sorting of gene activities along tissue type is very robust and clearly visible at a low baseline of 1.5-fold up to 10-fold differential expression (Figure 2). When we consider the similarity of all gene expression values for the small subgroup of genes exhibiting ≥10-fold differential expression, we find that most tissue types generally maintain this self-sorting principle (Figure 2B). As we have discussed above, genes with ≥10-fold differential expression, including ECM components, MMPs, TIMPs, and several members of the keratin family, likely play conserved and vital roles in regenerative processes. However, their distinct expression profiles over the regenerative time course still allow for tissue type self-identification, suggesting that genes necessary for proper regeneration maintain tissue-inherent activities. Importantly, cluster analyses focused on genes with expression values between ≥1.5-fold and ≤2-fold produced results very similar to the ≥2-fold differential expression data set, suggesting that our ≥2-fold change requirement for differential expression can be considered a highly stringent baseline for this custom array analysis (Figure 2C).

**Figure 3:**
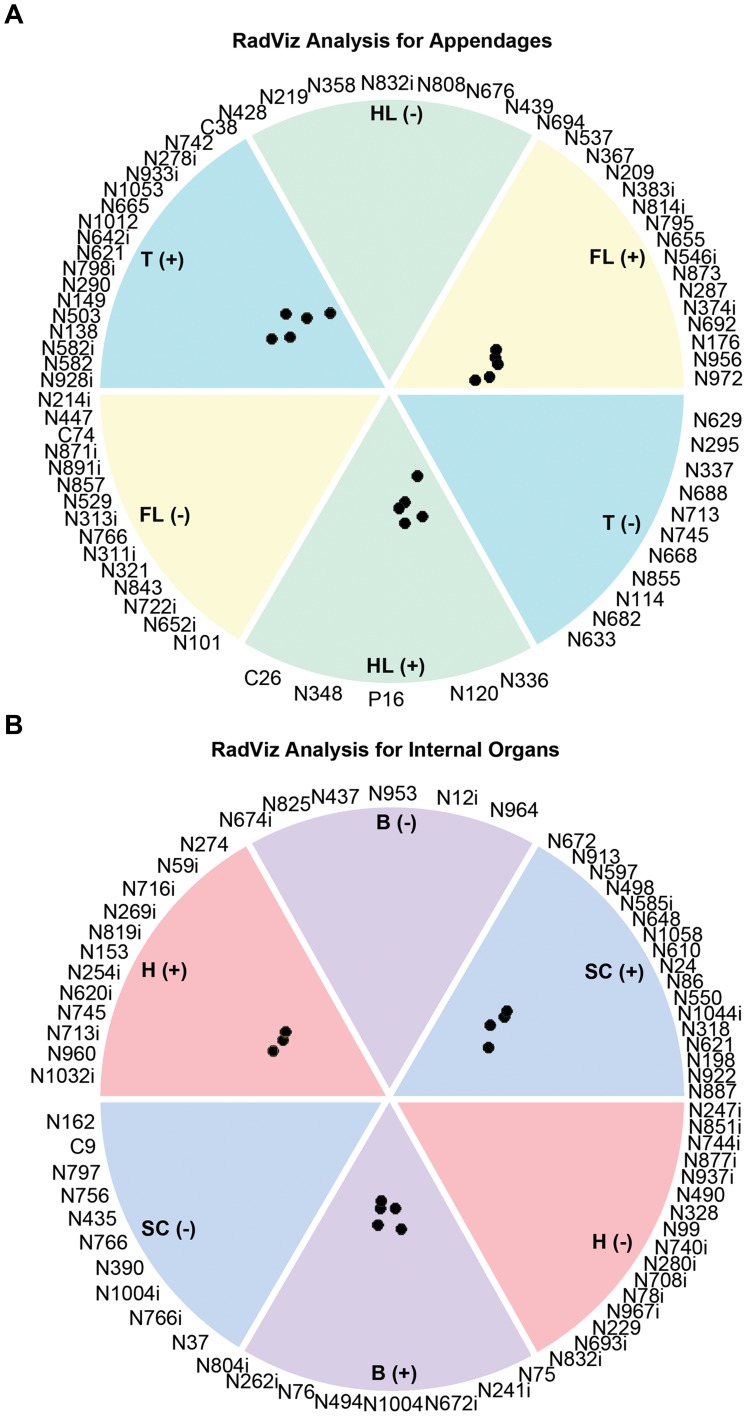
RadViz analysis identifies differential gene expression profiles that best segregate different regenerating tissue class members. The RadViz classification algorithm was used to classify three tissue classes to determine the top 90 genes (30 per class) that statistically best separate each tissue type class member from the other class members. Therefore, these specific genes’ differential activities most uniquely represent each regenerating tissue class type (full gene set with RadViz analysis available in Dataset S1). Gene activities most significant to the regeneration of each of the appendages (**A**) and each of the internal organs (**B**) are shown. The RadViz display circle is divided into equal sectors (or “pie slices”), two for each of the tissue type classes present in the analysis; the+sector and the – sector for each class both contain the most significant genes for that class that satisfy previously described criteria (Methods). The selected genes for each class are displayed within their class sector ranked in the order of their statistical power to carry out the class separation. The high degree of visual clustering of similar class member data points inside their appropriate sector, as well as the significant separation of members of each class from all other different class member points, indicates substantial accuracy using these genes, indicating that these specific genes can uniquely define distinct expression patterns within regenerating tissue types. Genes are listed by an abbreviated Probe ID; for example, N447 represents Nvg00447 (full gene set available in Dataset S2). Regenerating tissues are color-coded and abbreviated as: B = brain following spinal cord transection; H = heart; SC = spinal cord; T = tail; FL = forelimb; HL = hindlimb.

A deeper understanding of the specific genes that best define the regenerative process in each regenerating tissue can be achieved by utilizing RadViz classification and visualization [83]–[85]. The RadViz algorithm enables the identification of a defined subset of those gene activities that best distinguish a designated tissue class type from all other defined tissue classes in the analysis; it achieves this by using those gene expression values most uniquely and statistically different in those tissue classes. We employed the RadViz classification twice, first to determine the genes that best differentiate the regeneration of each of the three appendages, then to determine the genes that best differentiate each of the three internal organs. As Figure 3 illustrates, this novel approach identified 90 genes in each panel (30/tissue class) that distinguish the three tissue types with high statistical significance and define previously unreported tissue-specific regenerative activities (Dataset S2). We discuss a number of these below.

For example, comparing all the regenerating appendages, *myosin heavy polypeptide 13* segregated best with the forelimb, as this gene exhibited ≥2-fold upregulation in that appendage, while it was generally downregulated ≥5-fold in the hindlimb and tail. In contrast, several other muscle-related genes segregated best with the hindlimb tissue, including *troponin T3*, which was ≥10-fold downregulated in the regenerating hindlimb with less extreme differential expression in the other appendages, and *myogenic regulatory factor* (*MRF*) *5* (GenBank accession number X82838), which displayed a more substantial downregulation in the hindlimb relative to the regenerating forelimb and tail. It seems plausible that this more extreme downregulation of muscle-related genes could play an important role in the dedifferentiation of the regenerating hindlimb, given the larger muscle mass of this tissue type. RadViz analysis for the regenerating appendages also revealed the divergent use of certain growth factors during early regeneration. In particular, *fibroblast growth factor* (*FGF*) *2* and *connective tissue growth factor* (*CTGF*; GenBank accession number AJ271167) did not exhibit differential regulation in the regenerating tail, but both genes were ≥2-fold downregulated during early limb regeneration.

Just as in the appendages, RadViz analysis of the regenerating internal organs revealed a range of tissue-specific gene activities. In comparisons of regenerating spinal cord, brain and heart, *tbx4* (GenBank accession number AF537187) segregated best with the spinal cord, as this gene exhibited ≥5-fold downregulation in that tissue type while it was not differentially expressed in either the regenerating heart or the brain following spinal cord injury. Similarly, *connexin 43* (GenBank accession number AB078503) segregated best with the regenerating spinal cord, with ≥2-fold upregulation, but no differential expression in other tissue types. *Thrombin* (GenBank accession number M81395), an established and critical player in urodele limb and lens regeneration [86], [87], segregated well with brain tissue during spinal cord regeneration, as it was ≥5-fold downregulated in this tissue type, but ≥2-fold upregulated in the other regenerating internal organs. In addition, we discovered that *syntaxin-7* identifies best with regenerating cardiovascular tissue, with a ≥2-fold upregulation in the heart compared to a ≥2-fold downregulation in both the brain and spinal cord.

### Internal Organ vs. Appendage Regeneration

**Figure 4:**
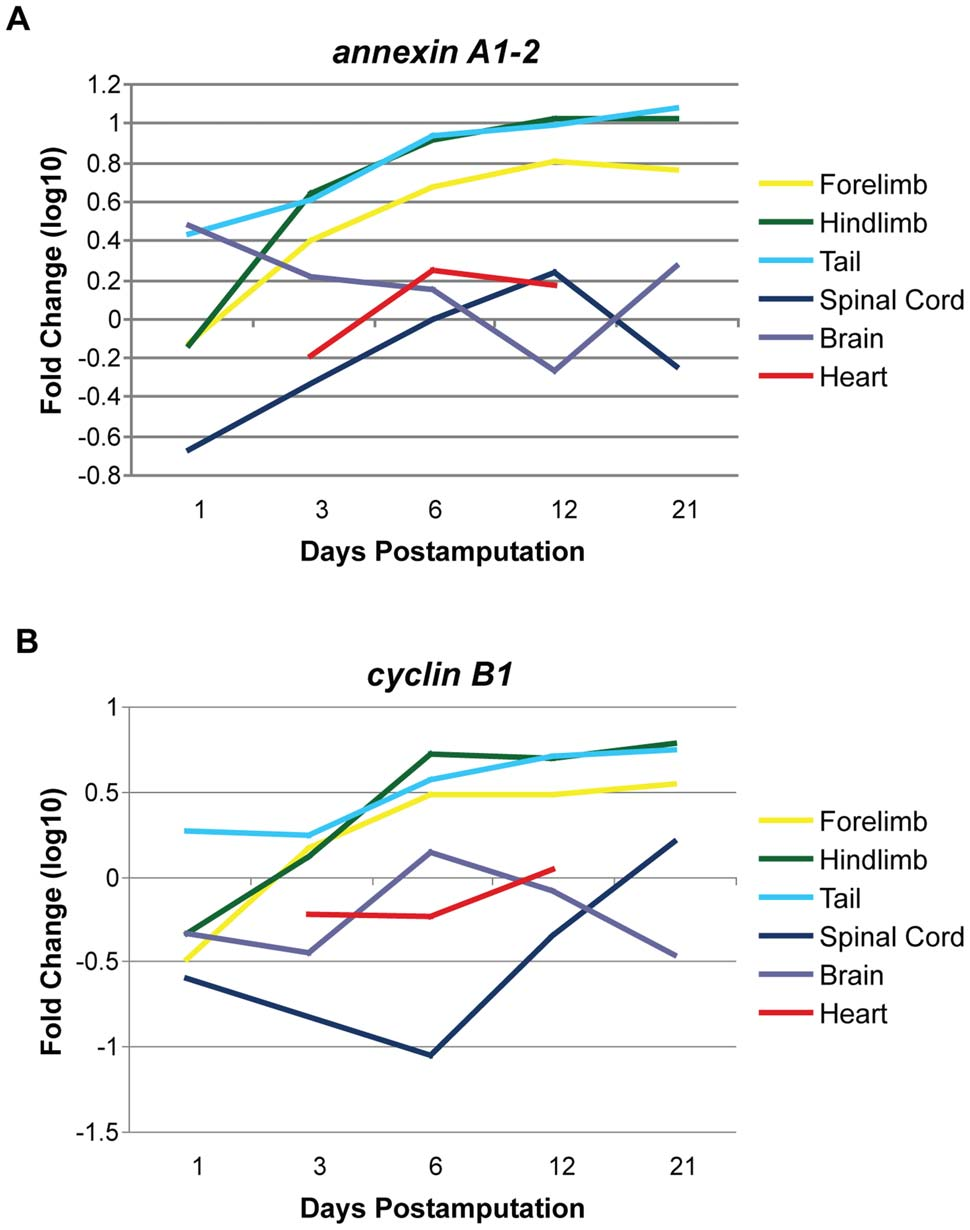
Immune response and cell cycle genes exhibit contrasting expression in regenerating internal organs and appendages. *Annexin A1-2* and *cyclin B1* are markedly upregulated during the first week of regeneration in the appendages, with both genes maintaining high expression through 21 dpa. In contrast, both *annexin A1-2* and *cyclin B1* are generally downregulated or not differentially expressed in the regenerating internal organs, suggesting divergent regulation of immune response and cell cycle in specific tissue types. Each time point was normalized to unamputated tissue (day 0) expression levels and log_10_ transformed. dpa = days postamputation. Note: Regenerating heart data was collected at 3, 7 and 14 dpa, but is graphically represented to align with the other tissue time points at 6 and 12 dpa for ease of comparison.

Annexin A1 is a member of a structurally related family of calcium- and phospholipid-binding proteins and has been implicated as a downstream mediator of anti-inflammatory glucocorticoids [88]–[91]. In the context of regeneration, the upregulation of annexin A1 could serve as a mechanism to limit the inflammatory response by restricting pro-inflammatory processes. Importantly, annexins may also be involved in the modulation of kinase activities in signal transduction, the maintenance of cytoskeleton and extracellular matrix integrity, and tissue growth and differentiation, which would indicate additional non-immune functions in regenerative processes [92], [93]. Our *annexin A1-2* expression profiling reveals a substantial upregulation, gradually reaching a plateau around 12 dpa in the forelimb, hindlimb and tail (Figure 4). Annexin A1 is also expressed at local lesions, possibly explaining why this gene is slightly upregulated in the spinal cord but gradually downregulated in the brain following spinal cord injury. The heart exhibits very low expression of *annexin A1-2*, possibly due to compensation by a different annexin family member [94].

Cyclin B1 is a regulatory protein involved in mitosis [95], and forced expression of this gene has been shown to induce proliferation [96], suggesting a possible role in bolstering progenitor cell growth during tissue regeneration. The forelimb, hindlimb and tail show a steady increase of *cyclin B1* (GenBank accession number AB005790) over the first 6 dpa, which correlates well with proliferation in tissues of regenerating forelimbs [42]. In contrast, *cyclin B1* is either downregulated or minimally active during early regeneration in the internal organs, possibly because of a cell proliferation delay in these tissues (Figure 4). In line with this notion, ongoing work in our laboratory shows that in regenerating newt hearts the majority of cardiomyocyte proliferation begins around 21 dpa (Mercer and Simon, unpublished data).

### Cardiac Muscle vs. Skeletal Muscle Regeneration

**Figure 5:**
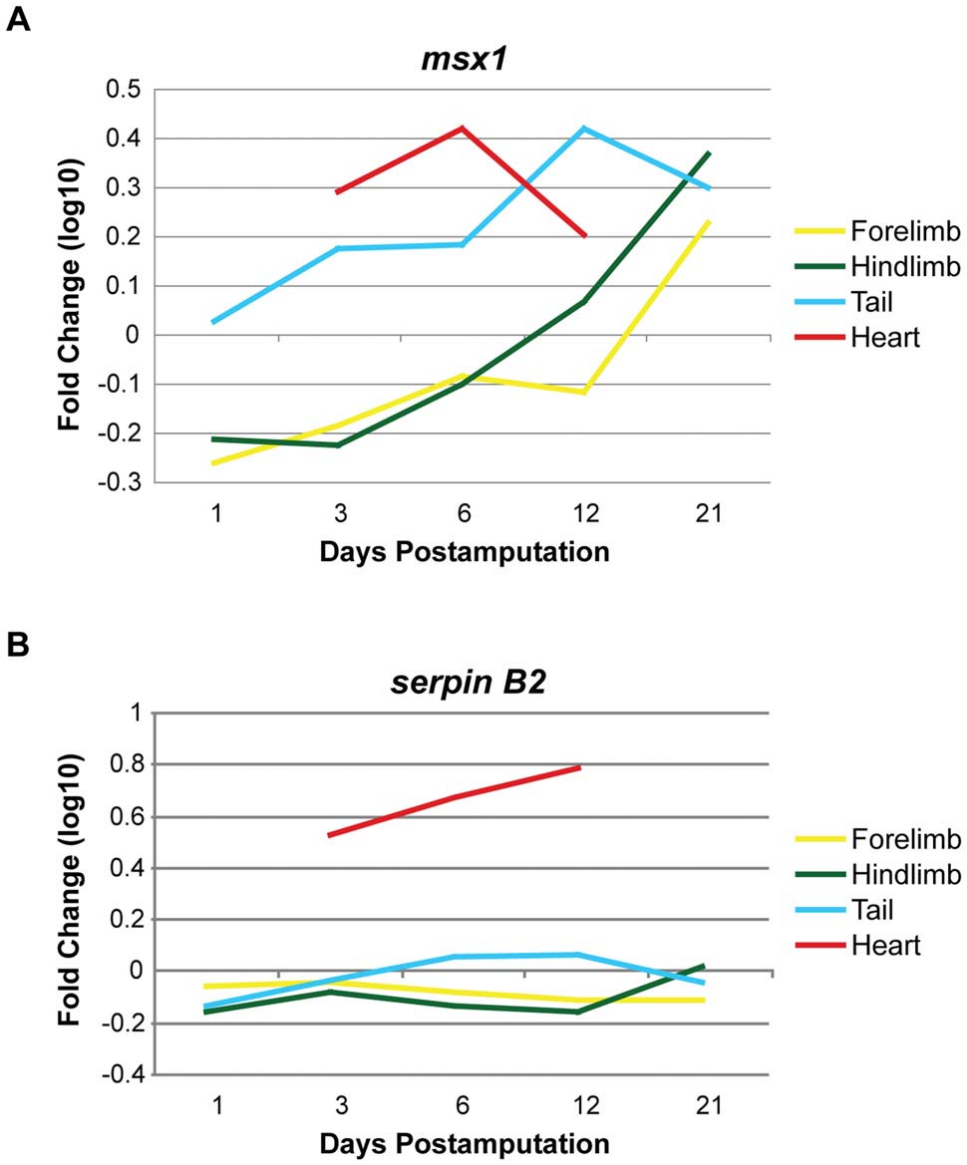
Immune response and pattern regulation genes display divergent expression in regenerating cardiac and skeletal muscle. Significantly upregulated during the early regenerative response in the heart, *msx1* expression peaks at 7 dpa during cardiac muscle regeneration. In contrast, *msx1* is not induced in regenerating appendages until 12 dpa (in the tail) or 21 dpa (in the hindlimb). Similarly, *serpin B2* is not differentially expressed in the regenerating appendages at any of the evaluated time points, while this gene is significantly upregulated throughout the first two weeks of heart regeneration. These opposing gene activities suggest deviations in the control of tissue patterning and inflammatory responses in regenerating cardiac and skeletal muscle. Each time point was normalized to unamputated tissue (day 0) expression levels and log_10_ transformed. dpa = days postamputation. Note: Regenerating heart data was collected at 3, 7 and 14 dpa, but is graphically represented to align with the other tissue time points at 6 and 12 dpa for ease of comparison.

The homeobox-containing transcriptional repressor *msx1* (GenBank accession number X82395) is a well-established key regulator of both appendage development and regeneration. In the developing limb bud, *msx1* expression demarcates the boundary between undifferentiated (*msx1* ^+^) and differentiating (*msx1* ^−^) cells [97], [98]. In addition to maintaining the undifferentiated state of the limb bud mesenchyme, msx1 may also regulate BMP signaling in the induction of the apical ectodermal ridge [99]. Robust *msx1* expression was shown in regenerating digit tips of the mouse [100] and a recent report revealed that an *msx1* ^+^ cell population might serve as a distal signaling center, essential for proper regrowth of the mammalian digit tip [101]. Msx1 is re-expressed in the early regeneration blastema of urodele limbs [102]–[104], zebrafish fins [105], [106], and *Xenopus* hindlimbs and tails [107]. Of note, in *Xenopus* development when tail regeneration cannot normally occur, the activation of msx1 can rescue regeneration during this refractive period [108]. Our current results find *msx1* induced in newt forelimb, hindlimb and tail blastemas (Figure 5), in context suggesting an important function for *msx1* in appendage regeneration. Interestingly, in line with *msxb* and *msxc* in zebrafish heart regeneration [109], our microarrays reveal that *msx1* is significantly upregulated during heart regeneration in the newt, suggesting that msx1 may not only maintain functionality in a variety of regeneration-competent species, but also in a number of regenerating tissue types (Table 2). Previous *in vitro* and *in vivo* data demonstrated that altering msx1 expression levels and/or functionality dynamically affects the differentiation status of skeletal muscle [107], [110], [111]. In light of our novel results, we speculate that *msx1* may define a subpopulation of progenitor cells and/or regulate cardiomyocyte differentiation during regenerative processes (Figure 5).

Serpin B2 is a protease inhibitor, acting as a repressor of the urokinase plasminogen activation system [112]. During regeneration, serpin B2 expression gradually increases in the heart, as higher levels of its anti-inflammatory activity may be necessary to prevent scar formation. As heart tissues must remain active and completely functional during regeneration, serpin B2 may serve as an important tissue-specific controller of inflammation and remodeling of regenerating cardiac muscle. In contrast, *serpin B2* exhibits very little differential activity during skeletal muscle regeneration, which may suggest that other anti-inflammatory mechanisms are utilized in the limbs, including annexins as discussed above.

### Spinal Cord vs. Brain vs. Tail Regeneration

When the spinal cord is injured through complete transection or severe crush injury, descending axons of supraspinal neurons are severed. In the newt, these severed axons regrow across the lesion and reestablish synapses that lead to functional recovery within 4 to 9 weeks of injury [113]. The nuclei of these neurons reside in the brain and therefore genes that are differentially expressed in the brain following spinal cord injury could have a determinant effect on spinal cord regeneration. Likewise, genes that are expressed at or near the lesion could dictate the regenerative cellular behaviors that occur at the site of injury. Therefore, we isolated RNA from both local injured spinal cord tissue and the brain following a transection injury.

**Figure 6:**
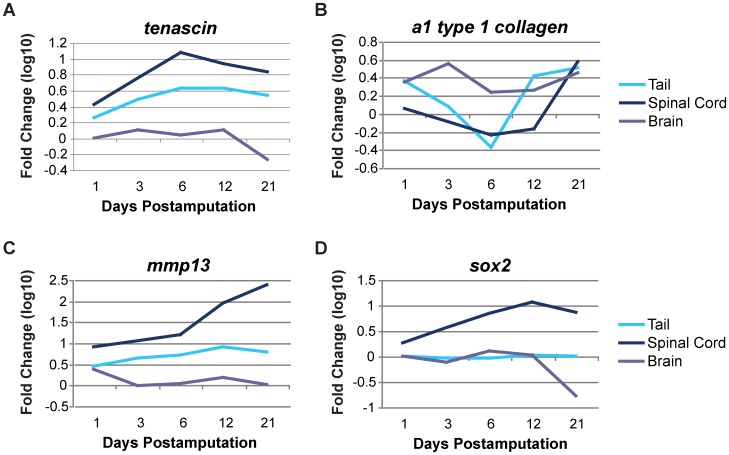
Extracellular environment and cell differentiation genes are differentially expressed during central nervous system regeneration. The extracellular matrix (ECM) component tenascin-C is steadily upregulated in both the regenerating spinal cord and tail, but is not differentially expressed in the brain. In contrast, *type I collagen* is upregulated during the early regenerative response in the brain following spinal cord transection, while it is initially downregulated at 6 dpa in both the regenerating spinal cord and tail with a subsequent, gradual upregulation. In line with this observed ECM remodeling, *mmp13* is significantly upregulated throughout early regeneration in the spinal cord and tail, but is generally not differentially expressed in the brain. Sox2, a key regulator of cellular differentiation status, exhibits substantial upregulation in the regenerating spinal cord, while it is not differentially expressed in the regenerating tail and is significantly downregulated in the brain at 21 dpa. Each time point was normalized to unamputated tissue (day 0) expression levels and log_10_ transformed. dpa = days postamputation.

Several genes exhibit differences in expression between the local tissues at the spinal cord lesion and the brain. Of particular interest are *tnc*, the *α_1_ chain* of type I collagen (GenBank accession number AB015438), *mmp13*, and *sox2* (GenBank accession number DQ490070). Each of these genes may play a specific role in the regenerative process and therefore their differential regulation is required for appropriate cellular responses (Figure 6).

TNC and type I collagen are extracellular matrix molecules that are inversely regulated during limb regeneration with apparently opposing roles. Our laboratory has recently reported that TNC carries signals that instruct cell behaviors during limb regeneration, such as myotube fragmentation, cell proliferation and migration [42], [48]. Type I collagen is often associated with differentiated tissue and accordingly is reduced in early regenerates, both by the downregulation of gene expression and by degradation of mature protein [27], [42]. Consistent with these results, in the region of the spinal cord lesion, collagen is downregulated while TNC is upregulated, such that it could similarly function to instruct progenitor cell behaviors in the spinal cord (Figure 6). This high TNC, low type I collagen matrix environment early in regeneration may be linked to the scarless healing of the spinal cord in the newt. Later, when axons are actively regrowing through the lesion [114], the *α_1_ chain* of type I collagen is re-expressed where it can participate in the production of type I collagen, a molecule that serves as a substratum for axon growth. In contrast, the brain shows very little differential expression of *tnc* and the *α_1_ chain* (Figure 6), reflecting the fact that the modulation of ECMs is only required where axon regrowth, cell proliferation and migration are actively occurring. Likewise, the expression of *mmp13*, a gene encoding a collagenase that targets intact triple helical collagens, is markedly upregulated within 1 day of injury in the spinal cord, but not the brain (Figure 6). These results suggest that, like limb regeneration, dynamic remodeling of the ECM at the lesion site is important for spinal cord regeneration.

*Sox2* is expressed in undifferentiated neuroepithelium and prevents the differentiation of neural progenitor and stem cells [115], [116]. *Sox2* is expressed in *GFAP-*expressing cells that line the ventricles in the newt brain [117]. The upregulation of *sox2* suggests that either the ependymoglial cells increase *sox2* expression or that there is an increase in the number of *sox2*-expressing cells in the region of the injury. An increase in expression within individual cells might suggest that the cells are dedifferentiating to become progenitor cells, while an increase in cell number would suggest a proliferative response. Given that *sox2* is upregulated 7-fold by day 6 and DNA synthesis as measured by EdU incorporation begins on day 6 or 7 (Figure S5), we think that dedifferentiation is a plausible explanation. Moreover, dedifferentiation has been suggested to occur in the urodele spinal cord [118]–[120]. The lack of differential expression of *sox2* in the brain is evidence that the production of progenitor cells in the brain is not required for the regenerative response (Figure 6).

*Sox2* exhibits little differential expression during tail regeneration (Figure 6). This might seem surprising given that the spinal cord must regenerate in synchrony with the tail and *sox2* is upregulated during spinal cord regeneration. However, the spinal cord retracts when cut and is likely not recovered in the harvesting of tail blastemas, especially at early time points. Furthermore, if it is present at all, the regenerating spinal cord is a very small proportion of the overall tissue mass in a regenerating tail blastema. Also, *sox2* is expressed at very low basal levels in the uninjured spinal cord and even when upregulated over 10-fold during spinal cord regeneration, it is still expressed at relatively low levels compared to many other genes, which could mask the *sox2* signal. Therefore, it is not surprising that *sox2* exhibits little differential expression during tail regeneration using our methods.

### What are the Major Biological Themes of Regeneration?

Gene term enrichment analysis is an important downstream task following microarray expression profiling, enabling a deeper understanding of the biological meaning of output gene lists [121]. Gene Ontology (GO) analysis (DAVID Bioinformatics Resource) enabled us to systematically map and identify the biological processes most relevant to the onset of regeneration, gaining a unique and unprecedented insight into natural regenerative processes at the molecular level [122].

We performed GO term enrichment analysis on annotated genes with ≥5-fold differential expression for at least one time point in at least one tissue type. This analysis revealed 43 significantly enriched terms for upregulated genes and 95 significantly enriched terms for downregulated genes (p≤0.01). As expected, enriched GO terms were found in each of the three main GO categories: cell component, biological process and molecular function; however, it is interesting to note that GO terms in the molecular function category seem underrepresented for the most significantly enriched terms, particularly for downregulated genes (full GO gene term enrichment analysis available in Dataset S3).

We found that our GO analysis for upregulated genes revealed a significant enrichment in cell components including extracellular matrices and cytoskeletal elements (Table 3). This result supports our previous gene expression work in regenerating limbs [27], [42]. As discussed above, a number of individual gene expression profiles strongly indicate that remodeling of both the extracellular environment and the intracellular cytoarchitecture is a critical and conserved regeneration-specific response. The GO analysis validates this conclusion and statistically highlights a number of genes that contribute to this reorganization. In addition, we discovered a significant enrichment of terms related to cytoskeletal organization, further supporting the conclusion that a reorganization of the cytoskeleton, with a specific upregulation of IFs, is a significant component of the regenerative response. The analysis also demonstrates that a number of terms related to developmental processes are significantly enriched for those genes that exhibit ≥5-fold upregulation in our arrays (Table 3). This finding agrees well with the specific gene activities highlighted above (Table 2) and also supports the overarching idea that regeneration reuses certain developmental programs to rebuild lost structures.

**Table 3:** Gene Ontology analysis reveals biological programs activated during vertebrate tissue regeneration.

GO Term	EASE Score	Associated Genes
**Cell Component**		
Extracellular region	1.34E−08	F11, WNT10A, BMP2, HDLBP, LGALS3, MMP9, TNC, MMP1, PGLYRP1, COL2A1, MMP3, MMP13,LGALS9, TIMP1, CXCL10, INHBA, ARG1, MMP10, FBLN2, CTGF, SERPINB2, COL1A1, FSHB, FN1
Intermediate filament cytoskeleton	2.10E−04	KRT5, KRT17, KRT15, KRT12, DST, NEFM
Muscle myosin complex	6.64E−04	MYH4, MYH13, TTN
Basement membrane	0.002901	TNC, COL2A1, FN1, TIMP1
Cytoskeleton	0.00348	CCNB1, KRT5, KRT17, ACTA2, KRT15, SPRR2B, KRT12, MYH4, MYH13, TTN, DST, NEFM
**Biological Process**		
Multicellular organismal catabolic process	4.78E−07	MMP10, MMP9, MMP1, MMP3, MMP13
Skeletal system development	7.65E−05	BMP2, LGALS3, CTGF, MMP9, COL2A1, COL1A1, MMP13, HOXD10
Cartilage development	1.89E−04	BMP2, CTGF, COL2A1, COL1A1, MMP13
Intermediate filament cytoskeleton organization	0.002925	KRT17, DST, NEFM
Cytoskeleton organization	0.006751	KRT17, TAGLN, CDC42BPA, TTN, DST, NEFM
Positive regulation of developmental process	0.007823	BMP2, KRT17, ID2, SOX2, JUNB
**Molecular Function**		
Structural molecule activity	2.50E−04	KRT5, KRT17, KRT15, KRT12, RPL10, COL2A1, COL1A1, TTN, NEFM
Endopeptidase activity	9.38E−04	F11, MMP10, MMP9, MMP1, SERPINB2, TTN, MMP3, MMP13
Calcium ion binding	0.003712	MMP10, FBLN2, PVALB, MMP9, MMP1, ANXA1, TTN, MMP3, DST, MMP13
Carbohydrate binding	0.006591	F11, LGALS3, CTGF, PGLYRP1, LGALS9, FN1

DAVID bioinformatics software was utilized to perform gene ontology (GO) term enrichment analysis for those annotated genes that exhibited ≥5-fold upregulation in at least one tissue type for at least one time point during the early regenerative response. Shown are the top 15 most significantly enriched, non-redundant GO_FAT terms associated with those genes. Terms are sorted by the EASE score, which represents a modified, more stringent Fisher Exact P-value. EASE scores ≤0.01 were considered significant.

Interestingly, GO term analysis for downregulated genes also revealed a significant enrichment in biological processes associated with development and embryonic morphogenesis (Table 4). While this finding may initially appear to be surprising, upon closer inspection these results further refine the idea that regenerative responses integrate activities proven in embryonic development. However, regeneration requires the construction of new tissue in context with pre-existing structures. Therefore, the fine-tuned spatiotemporal downregulation of specific developmental gene programs, particularly at the interface of old and new tissues, is an important aspect of natural regeneration. We also found that pattern specification processes are enriched among the downregulated genes (Table 4). One interpretation for this finding would be that the multiple tissues investigated in this GO analysis may have different temporal activities during early regeneration, such that some development- or pattern specification-related genes are downregulated only in certain tissues at specific time points, thus providing significant GO term enrichment results. Not surprisingly, the GO analysis demonstrates that biological processes including regionalization and cell fate commitment are significantly enriched among downregulated genes (Table 4). As our array data cover only the initial three weeks of regeneration, it is likely that molecular programs involved in cell and tissue differentiation are repressed during this time window, as the induction of regeneration largely centers on the generation and proliferation of stem-like progenitor cells. In support of this conclusion, the GO analysis indicates that cell components defining differentiated muscle are enriched in the downregulated gene set during the early regenerative dedifferentiation period.

**Table 4:** Gene Ontology analysis reveals biological programs that are decreased during vertebrate tissue regeneration.

GO Term	EASE Score	Associated Genes
**Cell Component**		
Sarcomere	4.67E−08	SYNE1, TCAP, NEB, PDLIM5, MYH4, MYH13, MYH6, TTN
Muscle myosin complex	8.94E−06	MYH4, MYH13, MYH6, TTN
**Biological Process**		
Pattern specification process	3.15E−08	MYF6, FGFR2, HES1, GSC, TCAP, HOXC5, OTX2, SIX3, RFNG, TTN, HOXD10, BMPR1A
Regionalization	3.20E−07	MYF6, HES1, GSC, TCAP, HOXC5, OTX2, SIX3, TTN, HOXD10, BMPR1A
Chordate embryonic development	1.62E−06	CCNB1, MYF6, FGFR2, HES1, GSC, TCAP, HOXC5, MYH6, TTN, NKX2-5, HOXD10, BMPR1A
Ear development	5.64E−06	FGFR2, HES1, GSC, TCAP, SOX2, OTX2, COL2A1
Sarcomere organization	1.25E−05	TCAP, NEB, MYH6, TTN
Sensory organ development	1.41E−05	FGFR2, HES1, GSC, TCAP, SOX2, OTX2, SIX3, COL2A1, NKX2-5
Respiratory system development	1.55E−05	FGFR2, HES1, CTGF, SOX2, TBX4, FGF2, BMPR1A
Adult heart development	2.16E−05	TCAP, MYH6, TTN, NKX2-5
Embryonic morphogenesis	2.22E−05	FGFR2, HES1, GSC, TCAP, SOX2, OTX2, TBX4, COL2A1, HOXD10, BMPR1A
Skeletal system development	2.98E−05	FGFR2, GSC, LGALS3, CTGF, HOXC5, TBX4, COL2A1, HOXD10, BMPR1A
Cell fate commitment	4.07E−05	FGFR2, HES1, SOX2, OTX2, SIX3, FGF2, HOXD10
Embryonic organ development	7.87E−05	FGFR2, GSC, TCAP, SOX2, OTX2, COL2A1, HOXD10, BMPR1A
Positive regulation of cellularbiosynthetic process	1.18E−04	HSP90AB1, MYF6, HES1, INHBA, SOX2, OTX2, IRF1, SIX3, NKX2-5, FGF2, HOXD10

DAVID bioinformatics software was utilized to perform gene ontology (GO) term enrichment analysis for those annotated genes that exhibited ≥5-fold downregulation in at least one tissue type for at least one time point during the early regenerative response. Shown are the top 15 most significantly enriched, non-redundant GO_FAT terms associated with those genes. Terms are sorted by the EASE score, which represents a modified, more stringent Fisher Exact P-value. EASE scores ≤0.01 were considered significant.

### New Hypotheses for Regenerative Biology

Our integrated approach of employing differential display and corresponding microarrays has enabled the identification of specific gene programs with potential critical functions in the induction and maintenance of regenerative processes in five different tissue types (Figure S1). Specifically, the pre-selection of cDNAs printed on our arrays produced a significantly enriched regenerative gene set, which allowed for extensive qualitative and quantitative analyses. In addition to uncovering conserved molecular programs activated in vertebrate regeneration, our hierarchical clustering also revealed that different regenerating tissues exhibit tissue-specific gene activity profiles. In light of the presented results, we hypothesize that gene activities related to immune response, tissue patterning, extracellular remodeling, cytoskeletal rearrangements and cell signaling synergistically support regenerative growth across multiple tissue types. We further propose that remarkably similar gene groups are expressed in slightly different patterns and at different levels during the regeneration of distinct tissue types, consequently establishing tissue-specific regenerative programs. Our data support a model in which the early stages of regeneration require remodeling of intracellular architecture, in a process that likely includes specific communication with the extracellular matrix and which proceeds through the generation of stem-like progenitor cells, proliferation, and the onset of patterning cues. Collectively, these results highlight the emerging theory that regenerative processes reutilize a number of developmental programs, while also recognizing the modifying principle that regeneration is not simply reactivated development.

We also note that we have identified several transcripts that demonstrate significant upregulation in each of the investigated tissue types that, as of yet, cannot be placed into any known gene family. The reason for this may lie in the nature of our differential display products used for printing the arrays, as these cDNAs are biased for 3′-UTRs. As indicated in Figure S1, we have performed hundreds of RNA Ligase Mediated-RACE (RLM-RACE) reactions on these so-called orphan genes to extend sequence reads 5′ and increase our chances of identifying orthologs or gene families. Interestingly, despite an overall success rate of approximately 65% in the RLM-RACE reactions, in many cases significant homology to annotated genes or open reading frames could not be detected in the longer sequences. Given that RLM-RACE is designed to amplify exclusively full-length transcripts, we can draw one of the following conclusions: 1) a substantial number of these differentially expressed transcripts encode very small peptides that play some yet unknown role in regeneration; or 2) these transcripts do not encode any protein and have an alternative function, e.g. regulation of other genes through gene silencing or some other mechanism. An intriguing possibility would be that microRNAs (miRNAs), rather than proteins, are encoded by these sequences. miRNAs are now widely regarded as regulators of gene expression in development and disease, and several recent studies have revealed that the interactions of miRNAs and their targets play a significant role in the regenerative response of both newts and zebrafish [123]–[127]. Future research will test the possibility that these orphan genes represent miRNAs with unexplored functions in vertebrate regeneration.

Our new findings represent a substantial step forward in understanding the molecular basis of vertebrate regeneration, providing new avenues of research in regenerative biology. The identification of overarching, regeneration-specific genes that are conserved across various tissue types will enable new opportunities to induce regenerative responses. These genes and the corresponding pathways they function in should be considered key targets for future investigations in both regeneration–competent and –incompetent species. However, these common molecular programs must be viewed in light of our discovery that regenerating tissues exhibit a remarkable degree of tissue-specific gene activities, and the integration of these tissue-inherent molecular programs may require the development of distinct protocols to stimulate regenerative repair of different tissues. Transferring this knowledge from the newt to mammalian models may ultimately enable the development of regenerative therapeutic treatments in humans.

## Materials and Methods

### Animal Care and Surgical Procedures

All animal procedures were approved by the Institutional Animal Care and Use Committees at either the University of Utah or Northwestern University. Adult newts (*Notophthalmus viridescens*) were purchased from either Charles D. Sullivan Company, Inc. (Nashville, TN) or Connecticut Valley Biological Supply Company (Southampton, MA) and housed at 22°C in tanks containing filtered dechlorinated water. Newts were fed live California blackworms every three days.

Newts were anesthetized by submersion in neutralized 0.1% w/v ethyl 3-aminobenzoate methanesulfonate salt (Tricane) for 10–15 minutes and then placed on ice for 15–20 minutes. Limbs were amputated through the stylopod at the midpoint of the humerus or femur and a ∼1.5 mm piece of the stylopodial region of the removed portion of the limb was immediately flash frozen in liquid nitrogen. Tails were amputated 2 cm distal to the hindlimbs and a ∼1.5 mm piece of the removed tail adjacent to the amputation site was flash frozen. Spinal cords were transected 1 cm cranial to the hindlimbs as described previously [114]. An equivalent section of uninjured spinal cord and the brain were collected from another set of newts and each of these tissues was flash frozen. The apex of the heart (10–20% of the ventricle) was removed with iridectomy scissors and immediately flash frozen. All frozen tissues were stored at −80°C and used as intact/uninjured (day 0) controls. Entry wounds for the spinal cord transections and heart ventricle amputations were closed by suture, surgical glue or secondary intention and regeneration was allowed to proceed.

At the specified time periods, regenerating tissues were collected, flash frozen in liquid nitrogen, and stored at −80°C until RNA extraction. Approximately 1.5 mm of regenerating limb or tail tissue was harvested from the stump of the regenerate. A 4 mm piece of the regenerating spinal cord was collected that encompassed both the lesion and 2 mm of additional tissue cranial and caudal to lesion. The entire brain from each newt with a spinal cord injury was also collected. The lower one-third of the ventricle containing the amputation plane was collected as heart regenerates. All newts were sacrificed at the time of tissue collection.

### RNA Extraction

Tissues from 3 to 10 regenerates at each time point were pooled and RNA was extracted from regenerating and control tissues using the phenol-free Ambion RNAqueous-4PCR Kit, which utilizes guanidine thiocyanate and glass fiber filters to isolate purified RNA. Concentration of the RNA was determined using a NanoDrop spectrophotometer (Thermo Scientific, Wilmington, DE, USA) and RNA integrity was assessed using a Bioanalyzer 2100 (Agilent, Santa Clara, CA, USA).

### Microarray Construction and Hybridization

Two custom microarrays were prepared by Agilent Technologies using data from both in-house DNA sequences and public databases. The initial microarray (Microarray A) represented 1,876 putative transcripts from 6 newt species with 93% of the transcript sequences derived from *N. viridescens*. A 60-mer oligonucleotide was designed for each transcript using Agilent eArray software and each gene template was represented 23 times on a grid containing 44,450 oligonucleotides. As part of the 44,450 oligonucleotides, 1,417 Agilent control probes (positive, negative, and ratiometric) were also included to assess the quality of the hybridization. For the heart regeneration studies, a subsequent microarray (Microarray B) representing 1,915 putative transcripts from 10 newt species was also constructed. This microarray contained all probes present on Microarray A, with additional, newly acquired sequences. Over 90% of *N. viridescens* genes in both arrays were cloned from mRNA isolated from regenerating tissues during differential display analysis [25]–[27]. In some cases, it was unknown which strand was the coding strand; therefore, a set of probes was generated that could hybridize to both strands. These probes have the designation “i” to indicate the inverted or complementary strand. For example, Nvg00415i is the complementary strand to Nvg00415. Standard BLAST searches using NCBI databases were performed to identify gene orthologs or most closely related homologs when possible.

RNA was isolated as described above from regenerating limbs, tails and brain tissues (following spinal cord transection) at 0, 1, 3, 6, 12, or 21 dpa, regenerating spinal cords at 0, 1, 6, 12, and 21 dpa, and regenerating hearts at 0, 3, 7, and 14 dpa. All probe preparation, hybridization, and quality control measures were done by the Huntsman Cancer Institute Microarray Core Facility at the University of Utah. RNA probes were generated by amplifying the isolated RNA and labeling with Cy3 or Cy5. Microarrays were competitively hybridized with Cy5- and Cy3-labeled probes from regenerating and day 0 (intact) tissues. Microarray A was used for the hybridization of all RNA probes, except those prepared from heart tissue. Microarray B was used for RNA probes generated from heart tissue. Following hybridization, the quality reports for each microarray were examined to ensure uniformity of median signal intensities, to determine the number of bad features on each array, and to confirm that ratiometric controls performed as expected. All bad features were eliminated from further analysis (bad features typically represented <0.1% of all spots on the array). Importantly, no microarray approached the standard facility threshold (≥1% bad features) for the elimination of all data generated from that microarray. The microarrays were Lowess normalized, the Cy5:Cy3 ratio (regenerating vs. intact tissues) for each hybridization was log_10_ transformed, and the geometric means and standard deviations were obtained for repetitive hybridizations. All microarray experiments conform to MIAME guidelines.

### Quantitative RT-PCR

Regenerating (3 dpa) and control tissues from 15 to 30 newts were pooled, RNA extracted using Ambion ToTALLY RNA kit (Life Technologies, Grand Island, NY, USA), DNase treated using the Ambion TURBO DNA-free kit and concentration determined with a NanoDrop spectrophotometer (Thermo Scientific, Wilmington, DE, USA). Reverse transcription of total RNA was performed according to the manufacturer’s instructions using SuperScript III Reverse Transcriptase with oligo dT primers (Life Technologies). Primers were validated for the PFAFFL method [128], with efficiency values derived for every primer pair from at least 2 unique biological samples with 3 technical replicates each. Specific gene primers were designed employing Integrated DNA Technologies PrimerQuest and OligoAnalyzer online tools and described in Table S2. Real-time PCR was performed in triplicate on a Bio-Rad iQ5 Cycler using the iQ SYBR Green Supermix (Bio-Rad) for detection of amplified DNA. Amplification conditions included an initial 3-min denaturation step at 95°C followed by 45 cycles of 95°C for 20 seconds, 57°C for 20 seconds, and 72°C for 30 seconds. Data were analyzed using the PFAFFL method and normalized to the control gene *histone acetyltransferase 1*. *Histone acetyltransferase 1* was selected from a group of 10 genes that had exhibited the least variation between time points based on microarray analysis. Normalized values were converted to relative values by using the corresponding intact tissues as the calibrator. Average fold changes were log_10_ transformed and standard deviations calculated in Excel.

### In Situ Hybridization

All steps were performed at room temperature (RT) unless otherwise specified. Intact and 6-day regenerating hindlimbs and intact and 12-day regenerating forelimbs were harvested from anesthetized animals. Spinal cord tissues that flanked each side of the lesion by approximately 2 mm were collected at 12 days post transection. Tissues were fixed with 4% paraformaldehyde (PFA) in PBS overnight at 4°C, and rinsed with PBS. Limbs were decalcified with Morse’s solution (22.5% formic acid, 10% sodium citrate) for 24 hrs, rinsed with PBS, dehydrated in a series of solutions containing increasing concentrations of ethanol (50%, 70%, 95%, 100%, 30 min each; 100%, 1 hr), infiltrated with paraffin (75% Hemo-De in EtOH, 20 min; 100% Hemo-De, 1 hr; paraffin, 1 hr, 60°C, 15 Hg vacuum), and embedded in paraffin. Sections 10–12 µm thick were mounted onto slides, left on a 37°C slide warmer overnight, and stored at 4°C. Prior to ISH, sections were dewaxed (Hemo-De, 10 min, 2 times; 75% Hemo-De in EtOH, 5 min; 100% EtOH briefly, then 3 min) and rehydrated into PBS (95% EtOH, 3 min; 70% EtOH, 3 min; PBS, 5 min). Sections were then post-fixed with 4% PFA in PBS for 15 min, washed with PBS for 5 min, quenched of endogenous peroxidases with 1% H_2_O_2_ in PBS for 30 min, washed with PBS for 5 min, and permeabilized with 5 µg/ml Proteinase K in TE (100 mM Tris-HCl, 50 mM EDTA, pH 8.0) for 15 min at 37°C. Sections were washed with PBS for 5 min, briefly equilibrated in TEA (0.1 M triethanolamine, pH 8.0), acetylated with 0.25% acetic anhydride in TEA for 15 min, washed in PBS for 5 min, and pre-hybridized in hybridization buffer (4X SSC, 50% formamide, 1X Denhardt’s solution, 200 µg/ml yeast RNA, 500 µg/ml heat denatured herring sperm DNA, 10% PEG6000) for 30 min at 55°C. Digoxigenin labeled riboprobes were diluted in hybridization buffer to 500 µg/ml, heat denatured for 3 min at 80°C, and added to the sections. Sections were hybridized in a humidified box overnight at 55°C. After hybridization, sections were briefly washed at 55°C in wash solution (4X SSC, 50% formamide, 1X Denhardt’s solution) and then for an additional 10 min. Subsequent washes included a series of solutions with lowering concentrations of wash solution (75% wash solution/25% 2X SSC, 50% wash solution/50% 2X SSC, and 25% wash solution/75% 2X SSC) for 10 min each, two washes with 2X SSC for 20 min each, two washes with 0.2X SSC for 20 min each, and then two washes at RT in MABT (100 mM maleic acid, 150 mM NaCl, 0.1% Tween-20, pH 7.5) for 10 min. Sections were then blocked with MABTB (MABT with 1% BSA and 10% heat inactivated horse serum) for 1 hr, incubated overnight at 4°C with anti-DIG-POD (HRP) antibody (Roche, 11207733910, 150 U/ml) diluted 1∶1000 in MABTB, rinsed five times with MABT for 10 min, rinsed with TNT (100 mM Tris-HCl, 150 mM NaCl, 0.05% Tween-20, pH 7.5) for10 min. The signal was then amplified by incubating the sections in tyramide-Cy5 (PerkinElmer, NEL745001KT) diluted 1∶50 in diluent for 20 min. Sections were washed 5 times with TNT for 10 min, incubated with SYTOX green nucleic acid stain (Invitrogen, S7020) diluted 1∶50,000 in 2% in DMSO in TNT for 15 min, washed with TNT, then 50% TNT, then water for 5 min each wash. Sections were mounted with Fluoromount G (Fisher, OB100-01) and coverslips were sealed with clear nail polish. Sections were imaged on an Olympus FV300 laser scanning confocal microscope using a 10X or 20X air objective. Images were processed with Image J, version 1.40 g (NIH, Bethesda, MD) and Adobe Photoshop CS2. Levels were adjusted in Photoshop to maximize the signal to noise ratio.

### EdU Staining

Newt spinal cords were injured by transection at the 8^th^ vertebra (lower thoracic region). Six hours before sacrifice, newts were injected intraperitoneally with 100 µl of 500 µM EdU (Life Technologies). The tissues were harvested at the time of sacrifice and serial cryosections of 16 µm were collected. Sections were fixed in 4% PFA for 5 min, rinsed in PBS, permeabilized with 0.5% Triton X-100 in PBS for 1 min then rinsed with PBS. Sections were blocked for 30 min in blocking buffer (20% goat serum, 0.2% bovine serum albumin, 50 mM ammonium chloride, 25 mM glycine, 25 mM lysine, and 0.02% sodium azide in PBS). To identify cells that incorporated EdU, sections were incubated for 30 min with AF488 conjugated azide (Life Technologies) diluted in 2 M Tris, 50 mM copper (II) sulfate and 0.5 M ascorbic acid. Primary antibodies were applied for 1 h and sections were rinsed with 0.1% Tween-20 in PBS. Slides were blocked again for 5 min before staining with the appropriate secondary detection reagents for 30 min. Slides were rinsed with 0.1% Tween-20 in PBS and mounted with FLUORO-GEL (Electron Microscopy Sciences, Hatfield, PA, USA).

### Cluster Dendrogram and Heatmap

The cluster dendrogram represents an unsupervised learning data mining technique employing a clustering algorithm that measures the similarity of all gene expression values considered in a starting data set. Relative similarity is represented via a tree structure, producing a hierarchical clustering where euclidean distance is the metric associated with tree branch point and limb features. Regenerating tissues that share similar gene expression profiles will have closer distances to a common branch point and be grouped into a cluster. Heatmaps provide a widely used 2-dimensional visual representation of microarray data ordered by the cluster dendrogram output. By assigning a color key to this data set, the relative probe intensity of each gene record for the different tissue types and time points can be visualized in different color intensities as a global view of differential gene expression, ordered into clusters. In this study, gene up-regulation fold changes are presented in shades of red, while down-regulation fold changes are presented in shades of green. All heatmap and cluster dendrogram visualizations in this study are implemented using the R software.

### RadViz Analysis

The RadViz algorithm was used to carry out supervised learning or classification, identifying those differential gene expression levels that best separated class members accurately into their distinct user identified classes in the data. As output, RadViz grouped together individual class members of the same identified classes (e.g., known tissue types) within localized regions (class sectors) of the visual output circle. Recognizing the best gene expression values to perform this classification out of the initial total gene expression set represents feature reduction of the original data. Therefore, the resulting classification of tissue types is based on the data of a specified selected number of genes. A detailed description of this software and its application to large biological data sets has been described elsewhere [83]–[85].

In brief, RadViz combines embedded clustering algorithms with statistically based feature reduction. Display of the algorithmic output is achieved using a high dimensional visualization. Data points (individual tissue arrays at a particular time postamputation) are placed on the 2-D RadViz circular display by a technique wherein the selected gene expression values are those that are statistically most significant at classifying the different tissue types. Classification results by positioning the same class member points into a common class area (+ or − class sector) of the RadViz display circle. Point positioning is carried out by the “force”, *f*, that any gene expression feature exerts on all the tissue data points in the RadViz display, where *f* is determined by a simple Hooke’s law relationship: *f = kd*. The spring constant, *k*, ranging from 0.0 to 1.0 is the normalized gene expression value for that sample, and *d* is the distance between the sample point and the perimeter point on the RadViz circle assigned to that feature. The final equilibrium placement of a sample point in the RadViz space is determined by the position where the total force is determined vectorially, from summing all gene expression value features to be 0. The gene expression value features are arranged in order around the RadViz circle arc (for that class sector) representing that particular class by the feature reduction and class discrimination algorithms to maximize the separation of the different identified classes of tissue points. Algorithmic feature reduction is based on the t-statistic with Bonferroni correction for multiple tests. The advantage of the RadViz technique is that an intuitive visual clustering of the results of the t-statistic based selection becomes immediately obvious upon viewing the visual output. The observed degree of ‘correct’ visual clustering together of class group points and their separation from all other class points generally correlates to the accuracy of any classifier built from the selected gene expression features.

In our specific use of RadViz, we have three classes and the RadViz output display circle is divided into six sectors. Each class has a+sector and a – sector across the circle from. In the two classification examples we present in this report, the top 90 genes (30/class distributed between+and − sectors) statistically were selected and used in the classification outputs we present. Gene expression values that are best at classifying those class points separately from the other class points meet the following criteria. For example, for class 1, selected genes whose average expression for class 1/(class 2+ class 3) >1 with a specific t-statistic criterion are shown placed in the+sector, while those whose average for class 1/(class 2+ class 3) <1 are shown placed in the – sector. A similar relationship applies to the genes placed in the other two class sectors.

### Gene Ontology Analysis

To extract biological features associated with large gene sets, we utilized the DAVID Bioinformatics Resource (the Database for Annotation, Visualization and Integrated Discovery) and its Gene Ontology (GO) term enrichment analysis. As described above, BLAST searches using NCBI databases were performed to identify gene orthologs or most closely related homologs to newt sequences when possible. Subsequently, the corresponding human/mouse official gene symbols were obtained for all annotated genes that exhibited ≥5-fold differential expression in the initial microarray analysis. These genes were uploaded to the functional annotation tool of DAVID utilizing the official gene symbol as the identifier on a mouse background, as no *N. viridescens* background exists in this software. GO annotation employed the GO FAT term set, which attempts to filter out the broadest GO terms such that more precise, biologically relevant terms can be acquired. The GO FAT term specificity is based on the number of child terms in a GO tree structure and is uniquely available through the DAVID Bioinformatics Resource [121], [129]. GO term enrichment was considered significant if the minimum number of genes associated with the corresponding term was ≥2 and the EASE score (a modified, more stringent Fisher Exact P-value) was ≤0.01. For the presented data sets (Tables 3 and 4), essentially identical GO terms (those with entirely or significantly overlapping associated genes) were omitted to avoid unnecessary redundancies; the entire GO data set is available in Dataset S3.

### Ethics Statement

This study was performed in strict accordance with the recommendations in the Guide for the Care and Use of Laboratory Animals of the National Institutes of Health. The protocol was approved by the Institutional Animal Care and Use Committees at either the University of Utah or Northwestern University (IACUC animal welfare assurance number A-3995-01; IACUC approval number 99015). All surgery was performed under anesthesia, and every effort was made to minimize suffering.

## Supporting Information

Figure S1 **Schematic of experimental design for integrated differential display and microarray analysis of regeneration.**
(TIF)

Figure S2 **Distribution of up- and down-regulated genes in multiple tissue types over a regenerative time course.** Representation of the number of differentially expressed genes in each tissue type at every evaluated time point during the initial three week regenerative response. Though gene upregulation is generally more prevalent, a substantial number of genes are downregulated in each tissue type at all time points. In addition, the number of up- and down-regulated genes follows a similar trend for each regenerating tissue over the time course. Regenerating tissue abbreviations: B = brain following spinal cord transection; H = heart; SC = spinal cord; T = tail; FL = forelimb; HL = hindlimb. Days postamputation are indicated following tissue type designation.
(TIF)

Figure S3 **Spatial expression patterns of** ***cxcl*** **mRNA during newt limb regeneration.** Longitudinal sections were imaged on a confocal microscope and a z-projection of 2–3 confocal planes was generated to represent the signal from the full-thickness of the limb. *cxcl* mRNA is shown in magenta and nuclei are shown in green. (**A)** *cxcl* is expressed at very low levels at 0 dpa (intact limb). (**B)** At 6 dpa, the proximal hindlimb (stylopod) expresses *cxcl* in the dedifferentiating mesodermal tissues (especially the muscle) of the limb stump. (**C)** At 12 dpa, the distal forelimb (zeugopod) expresses high levels of *cxcl* in the blastema, apical epithelial cap, and dedifferentiating muscle of the limb stump. (**D)** At 12 dpa, distal forelimb hybridized with a sense *cxcl* probe confirms the specificity of the signal. e  =  epithelium; m  =  muscle; b  =  bone; bl  =  blastema; aec  =  apical epithelial cap.
(TIF)

Figure S4 **Cellular expression patterns of** ***mmp9*** **in neural tissues following spinal cord transection.** (**A**) *mmp9* expression in the spinal cord at day 12 post-transection. A few cells express *mmp9* (green fluorescence, white arrows) in or near the lesion, but a greater number of cells express *mmp9* as the distance from the lesion increases. High levels of expression are seen between 100–650 µm from the lesion site. Ependymal cells begin expressing *mmp9* at a distance of about 275 µm from the lesion and exhibit high expression levels at 650 µm (white arrow). The white outline encloses the approximate area of the ependymal cells in the two panels on the lower left. (**B**) *mmp9* is expressed in gray matter cells of the newt hindbrain at day 12 following a thoracic spinal cord transection. AS = antisense probe; S = sense probe; SC = spinal cord.
(TIF)

Figure S5 **Cell cycle reentry during newt spinal cord regeneration.** (**A**) Cells in the intact newt spinal cord are quiescent. (**B–G**) Cross-sections of newt spinal cord 200–300 µm cranial to the lesion site. (**B**) At 5 days post-transection (Post-Tx), cells remain quiescent. (**C**) Ependymoglial cells have begun to reenter the cell cycle (arrowhead) by 6 days post-transection. (**D**) Cells of the ependyma, gray matter (arrowhead), and meninges (arrow) have reentered the cell cycle by day 7 post-transection. (**E–G**) Cells continue to proliferate through day 28 post-transection. Magenta staining = EdU incorporation signifying DNA synthesis; Blue = Hoechst 33342 staining of nuclei; Green = phosphotyrosine staining of cell membranes.
(TIF)

Table S1 **qRT-PCR validation of microarrays.** Representative genes from the microarray analysis were selected for qRT-PCR validation. qRT-PCR was performed with tissue samples at 3 days post-amputation (see Methods). Fold changes are derived from triplicate samples and presented as normalized log_10_ values. The respective fold changes of the microarray data at 3 days post-amputation are provided for comparison. Full array expression data is available in Dataset S1. Microarray and qRT-PCR data were generated from independent biological samples, but the overall correlation is 85%, with 44 of the 52 validations showing the same mode (up vs. down) of differential expression (≥2 fold change relative to control). In some cases, we note a wider dynamic range in the qRT-PCR expression levels, an observation previously reported for qRT-PCR validation assays [29]. FC = fold change; RT = qRT-PCR; SD = standard deviation.
(PDF)

Table S2 **Sequences of primers used for qRT-PCR.** PCR cycling conditions: initial denaturation at 95°C for 3 min; 45 cycles at 95°C for 20 s, 57°C for 20 s, and 72°C for 30 s.
(PDF)

Dataset S1 **Full array data for each regenerating tissue at all evaluated time points.**
(XLS)

Dataset S2 **RadViz algorithm classification outputs for best 30 genes and total genes discriminating regeneration in the appendages and internal organs.**
(XLS)

Dataset S3 **Enriched GO terms obtained from DAVID bioinformatics software for genes with ≥5-fold differential expression for at least one time point in at least one regenerating tissue type.**
(XLS)
