# Supplementary figures and images for: Multi-Tissue Microarray Analysis Identifies a Molecular Signature of Regeneration
Source: PLoS One. 2012 Dec 26;7(12):e52375. doi: 10.1371/journal.pone.0052375 (PMC3530543; doi:10.1371/journal.pone.0052375)

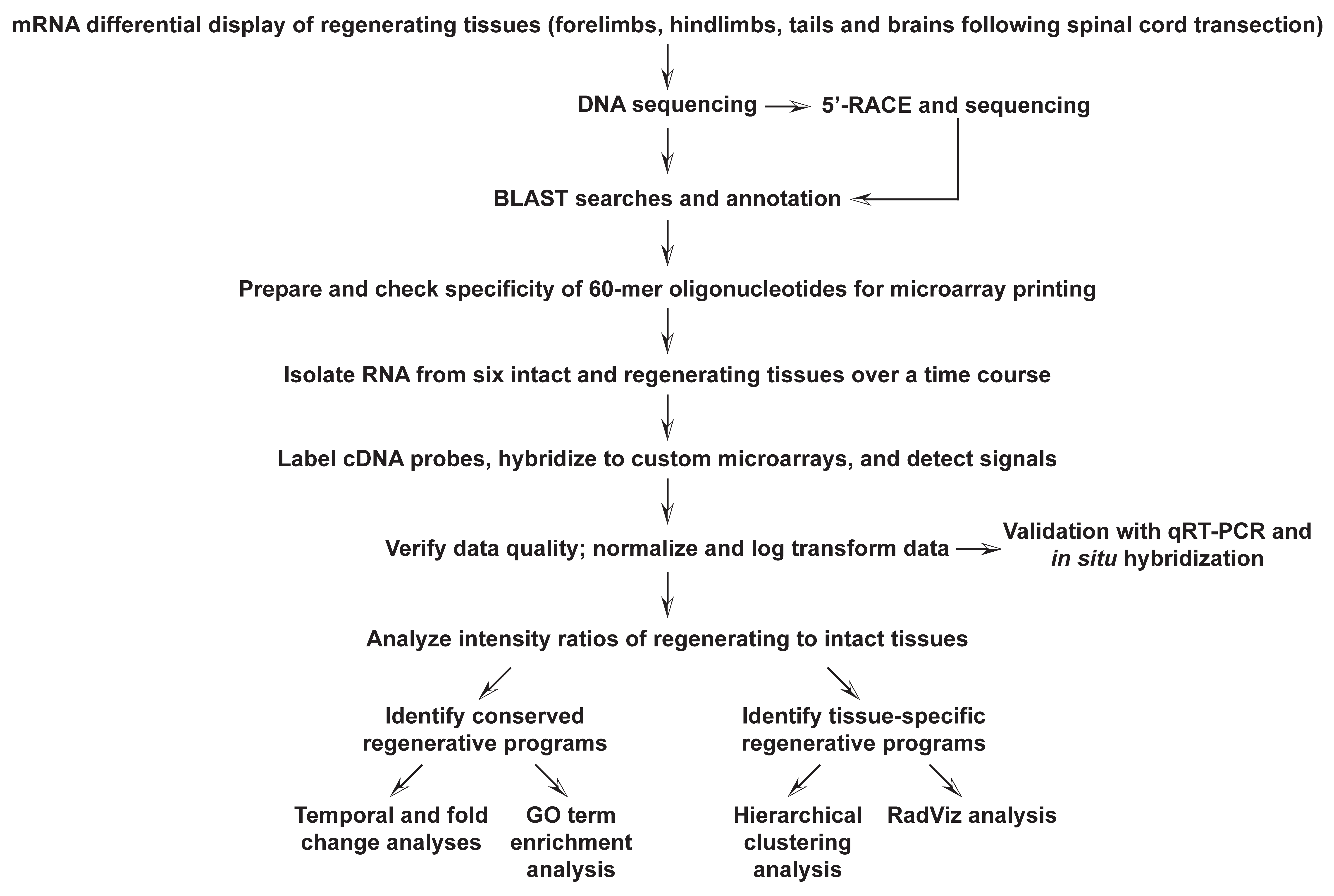

Supplement: Figure S1 — Schematic of experimental design for integrated differential display and microarray analysis of regeneration. (TIF) [file pone.0052375.s001.tif]

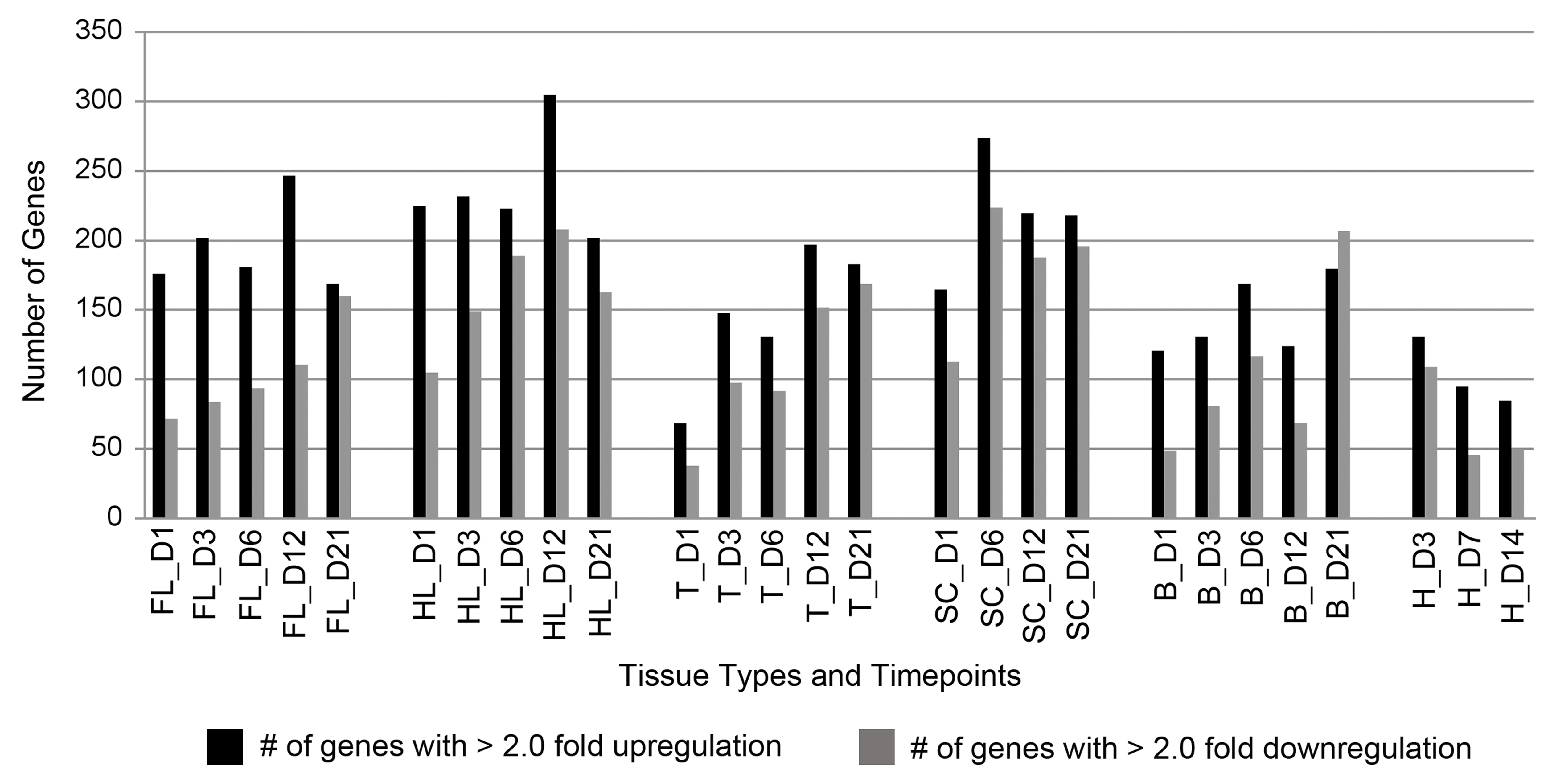

Supplement: Figure S2 — Distribution of up- and down-regulated genes in multiple tissue types over a regenerative time course. Representation of the number of differentially expressed genes in each tissue type at every evaluated time point during the initial three week regenerative response. Though gene upregulation is generally more prevalent, a substantial number of genes are downregulated in each tissue type at all time points. In addition, the number of up- and down-regulated genes follows a similar trend for each regenerating tissue over the time course. Regenerating tissue abbreviations: B = brain following spinal cord transection; H = heart; SC = spinal cord; T = tail; FL = forelimb; HL = hindlimb. Days postamputation are indicated following tissue type designation. (TIF) [file pone.0052375.s002.tif]

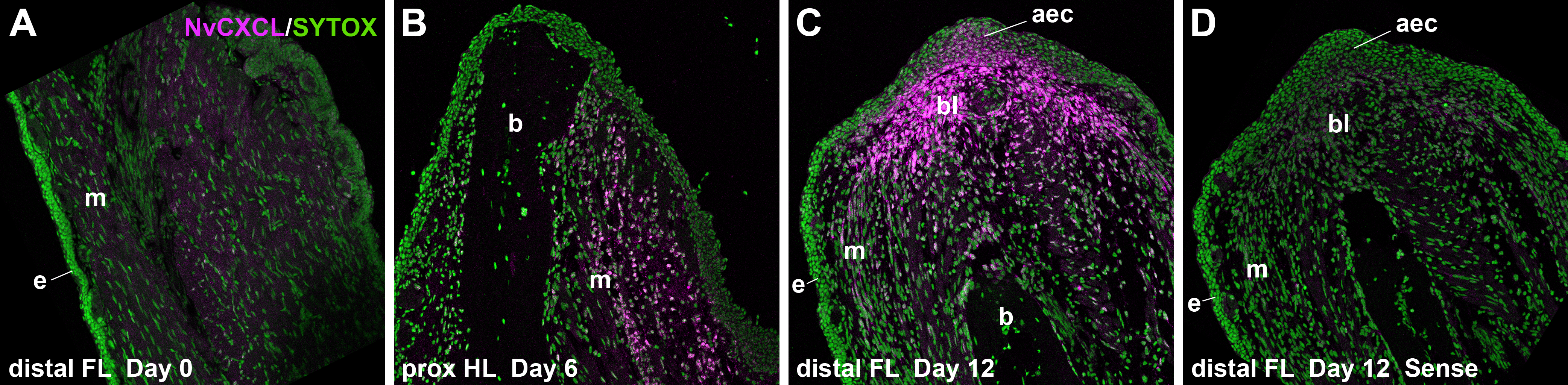

Supplement: Figure S3 — Spatial expression patterns of cxcl mRNA during newt limb regeneration. Longitudinal sections were imaged on a confocal microscope and a z-projection of 2–3 confocal planes was generated to represent the signal from the full-thickness of the limb. cxcl mRNA is shown in magenta and nuclei are shown in green. (A) cxcl is expressed at very low levels at 0 dpa (intact limb). (B) At 6 dpa, the proximal hindlimb (stylopod) expresses cxcl in the dedifferentiating mesodermal tissues (especially the muscle) of the limb stump. (C) At 12 dpa, the distal forelimb (zeugopod) expresses high levels of cxcl in the blastema, apical epithelial cap, and dedifferentiating muscle of the limb stump. (D) At 12 dpa, distal forelimb hybridized with a sense cxcl probe confirms the specificity of the signal. e = epithelium; m = muscle; b = bone; bl = blastema; aec = apical epithelial cap. (TIF) [file pone.0052375.s003.tif]

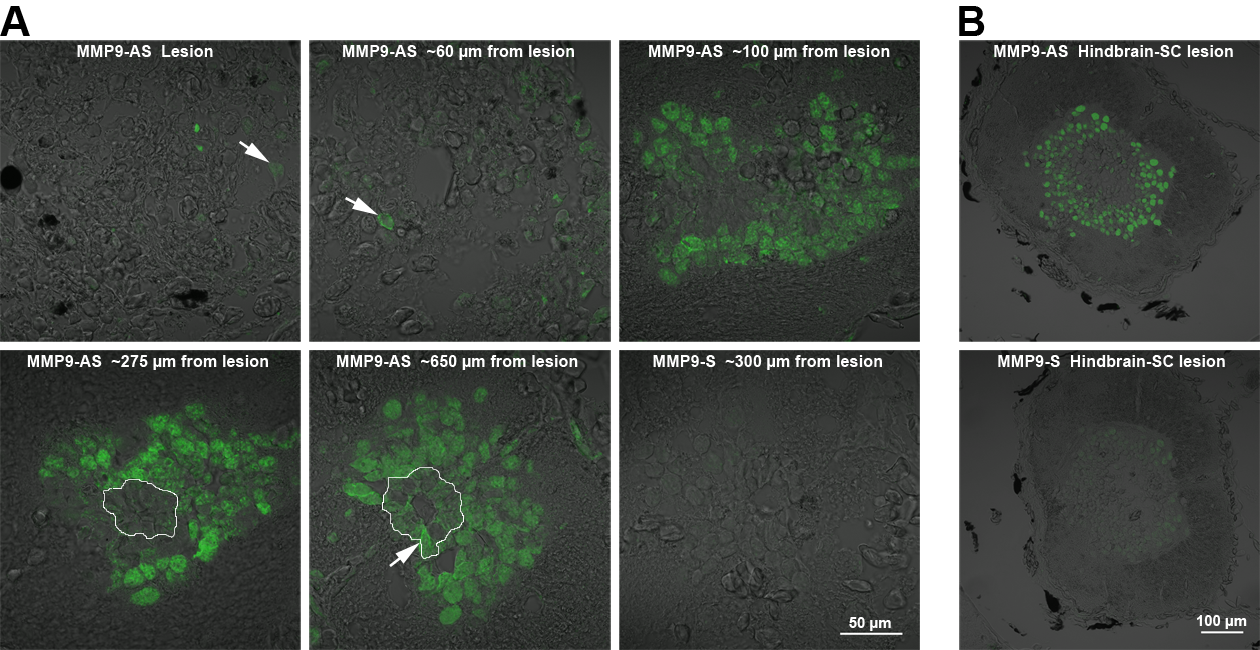

Supplement: Figure S4 — Cellular expression patterns of mmp9 in neural tissues following spinal cord transection. (A) mmp9 expression in the spinal cord at day 12 post-transection. A few cells express mmp9 (green fluorescence, white arrows) in or near the lesion, but a greater number of cells express mmp9 as the distance from the lesion increases. High levels of expression are seen between 100–650 µm from the lesion site. Ependymal cells begin expressing mmp9 at a distance of about 275 µm from the lesion and exhibit high expression levels at 650 µm (white arrow). The white outline encloses the approximate area of the ependymal cells in the two panels on the lower left. (B) mmp9 is expressed in gray matter cells of the newt hindbrain at day 12 following a thoracic spinal cord transection. AS = antisense probe; S = sense probe; SC = spinal cord. (TIF) [file pone.0052375.s004.tif]

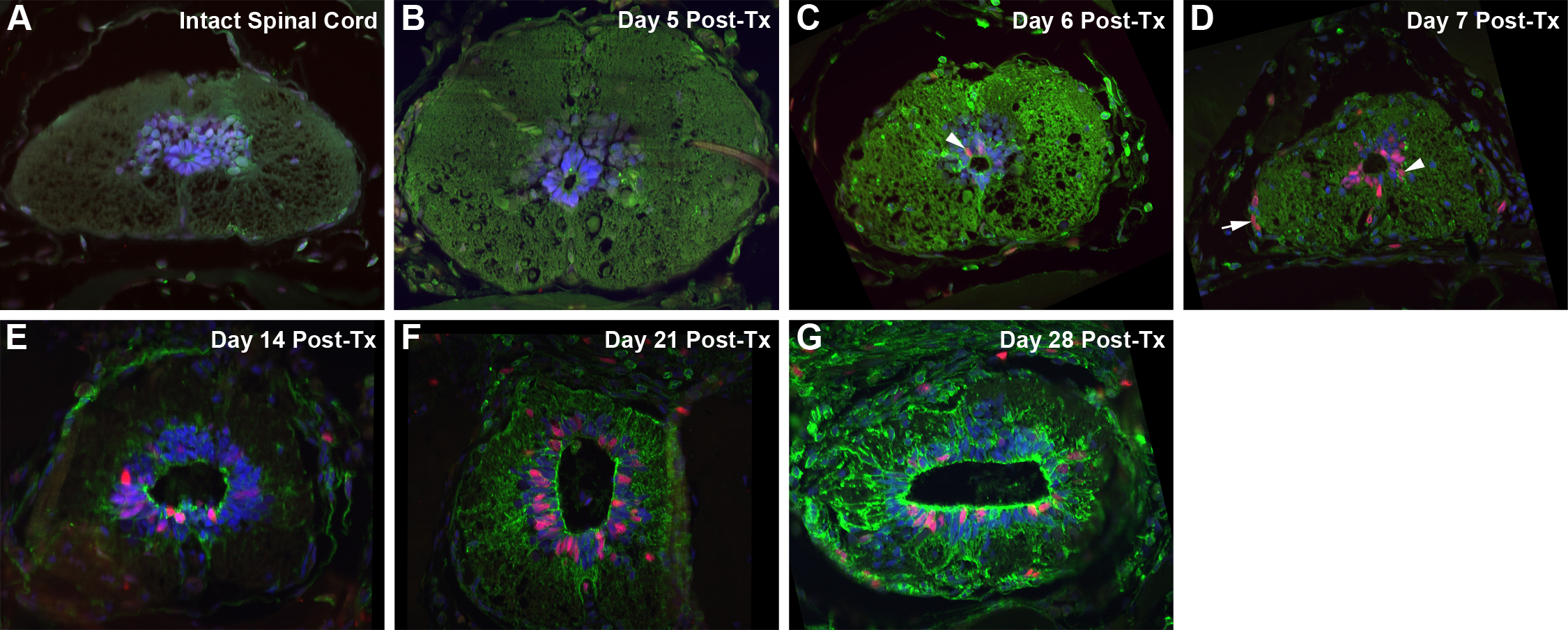

Supplement: Figure S5 — Cell cycle reentry during newt spinal cord regeneration. (A) Cells in the intact newt spinal cord are quiescent. (B–G) Cross-sections of newt spinal cord 200–300 µm cranial to the lesion site. (B) At 5 days post-transection (Post-Tx), cells remain quiescent. (C) Ependymoglial cells have begun to reenter the cell cycle (arrowhead) by 6 days post-transection. (D) Cells of the ependyma, gray matter (arrowhead), and meninges (arrow) have reentered the cell cycle by day 7 post-transection. (E–G) Cells continue to proliferate through day 28 post-transection. Magenta staining = EdU incorporation signifying DNA synthesis; Blue = Hoechst 33342 staining of nuclei; Green = phosphotyrosine staining of cell membranes. (TIF) [file pone.0052375.s005.tif]
