# Supplementary material for: Multi-Tissue Microarray Analysis Identifies a Molecular Signature of Regeneration
Source: PLoS One. 2012 Dec 26;7(12):e52375. doi: 10.1371/journal.pone.0052375 (PMC3530543; doi:10.1371/journal.pone.0052375)
Supplement: Table S1 — qRT-PCR validation of microarrays. Representative genes from the microarray analysis were selected for qRT-PCR validation. qRT-PCR was performed with tissue samples at 3 days post-amputation (see Methods). Fold changes are derived from triplicate samples and presented as normalized log10 values. The respective fold changes of the microarray data at 3 days post-amputation are provided for comparison. Full array expression data is available in Dataset S1. Microarray and qRT-PCR data were generated from independent biological samples, but the overall correlation is 85%, with 44 of the 52 validations showing the same mode (up vs. down) of differential expression (≥2 fold change relative to control). In some cases, we note a wider dynamic range in the qRT-PCR expression levels, an observation previously reported for qRT-PCR validation assays [29]. FC = fold change; RT = qRT-PCR; SD = standard deviation. (PDF) [file pone.0052375.s006.pdf]

GENE	Forelimb			Hindlimb			Tail			Heart		
	Array FC	RT FC	RT SD	Array FC	RT FC	RT SD	Array FC	RT FC	RT SD	Array FC	RT FC	RT SD
Apple4	0.425	0.738	0.024	0.831	1.367	0.033	0.543	0.873	0.033	0.847	0.714	0.023
Cyclin B1	0.159	0.031	0.054	0.105	0.134	0.011	0.236	0.087	0.033	-0.227	-0.485	0.031
Elafin1	0.753	2.005	0.037	0.924	1.345	0.026	1.118	1.476	0.039	1.580	1.272	0.019
FGF2	-0.428	-0.007	0.128	-0.435	-0.255	0.031	-0.050	-0.085	0.039	-0.228	-0.296	0.065
FGF2R	-0.253	-0.242	0.026	-0.183	0.012	0.038	-0.025	-0.127	0.064	-0.173	-0.388	0.044
Galectin9	0.911	1.260	0.012	1.034	1.281	0.028	0.557	0.301	0.019	0.197	0.030	0.026
Keratin17	0.309	0.451	0.097	0.503	2.655	0.024	0.684	1.759	0.072	0.900	0.555	0.062
MMP3/10a	0.303	1.863	0.045	1.573	1.903	0.036	0.660	1.514	0.038	1.065	1.126	0.072
TIMP1	0.618	1.051	0.015	1.006	0.974	0.022	0.840	1.078	0.031	0.752	0.406	0.054
Tenascin	0.246	0.980	0.091	0.246	0.930	0.028	0.477	0.861	0.032	0.631	0.853	0.086
Nvg00186	0.937	0.828	0.031	0.940	1.115	0.029	0.424	0.740	0.040	0.460	0.633	0.073
Nvg00195	0.666	0.956	0.023	1.262	1.525	0.031	0.841	1.170	0.058	0.262	0.147	0.006
Nvg00226	0.454	0.278	0.041	0.617	0.673	0.094	0.297	0.376	0.044	-0.043	0.241	0.057
