# Supplementary material for: Multi-Tissue Microarray Analysis Identifies a Molecular Signature of Regeneration
Source: PLoS One. 2012 Dec 26;7(12):e52375. doi: 10.1371/journal.pone.0052375 (PMC3530543; doi:10.1371/journal.pone.0052375)
Supplement: Table S2 — Sequences of primers used for qRT-PCR. PCR cycling conditions: initial denaturation at 95°C for 3 min; 45 cycles at 95°C for 20 s, 57°C for 20 s, and 72°C for 30 s. (PDF) [file pone.0052375.s007.pdf]

| Gene | 5'- Primer Sequence | 3'- Primer Sequence |
| --- | --- | --- |
| Apple4 | GCGGAATGCTGCCAGATTTAC | TGGGTCAACTGTGGACAAACG |
| Cyclin B1 | AAATGAAGTTCAGGCTGCTCC | TGGTGTAGGTGTGGTCAGTTAC |
| Elafin1 | CGTTAGAGGGGTAATTCGTAAGG | ATGGATTTGTTACTTGCTATTGGG |
| FGF2 | AGAAGAGCGACTCCTACATT | AGCCTGCCATCATCCTTCA |
| FGF2R | CGTGGCATTGAGAGGATGTTC | CCGTCGTTGTCGTCTTCATC |
| Galectin9 | TCGCCGTCTGAGAACATTGC | GCGTTTACTTTATGGAGCGGAATC |
| Keratin17 | CAAGGACGGGAAGGTTGTATCA | GGTTCTCTGGTCATCTCTTATGG |
| MMP3/10a | CAACACACTGGAAATGATG | TCAAATGGGTAGAAGTCAC |
| TIMP1 | CGGCGAATGTATTTGGGAGTC | ACAGGAGGTTGGAACAGATGG |
| Tenascin | CGACGAAGGATTCACAGGAG | CCATCATCACAGACACAGAGG |
| Nvg00186 | GATGCTGACGTGACCACTGG | CCTCTGGTCTACAGGACTATTACG |
| Nvg00195 | GCGGCAGACAGTAGAACTTAGC | CAGCAGGTGTAGAATCCCTCAAG |
| Nvg00226 | GCTAAAATGCCACACACAATACAG | TCGACTAAAAGCAGAAGTAGACAG |
| Histone acetyltransferase 1 | CGTGGAGGCTGATGATATTG | GCTCGCTGACTCAATGAAC |
